# Novel approach reveals genomic landscapes of single-strand DNA breaks with nucleotide resolution in human cells

**DOI:** 10.1038/s41467-019-13602-7

**Published:** 2019-12-20

**Authors:** Huifen Cao, Lorena Salazar-García, Fan Gao, Thor Wahlestedt, Chun-Lin Wu, Xueer Han, Ye Cai, Dongyang Xu, Fang Wang, Lu Tang, Natalie Ricciardi, DingDing Cai, Huifang Wang, Mario P. S. Chin, James A. Timmons, Claes Wahlestedt, Philipp Kapranov

**Affiliations:** 1 Institute of Genomics, School of Biomedical Sciences, Huaqiao University, 668 Jimei Road, Xiamen, 361021 China; 2 Department of Pathology, Second Affiliated Hospital of Fujian Medical University, Quanzhou, 362000 China; 3 Center for Therapeutic Innovation and Department of Psychiatry and Behavioral Sciences, University of Miami Miller School of Medicine, 1501 NW 10th Ave, Miami, FL 33136 USA; 4 Augur Precision Medicine LTD, Scion House, Stirling University Innovation Park, Stirling, FK9 4NF UK

**Keywords:** Genomic analysis, Genomics, DNA damage and repair

## Abstract

Single-strand breaks (SSBs) represent the major form of DNA damage, yet techniques to map these lesions genome-wide with nucleotide-level precision are limited. Here, we present a method, termed SSiNGLe, and demonstrate its utility to explore the distribution and dynamic changes in genome-wide SSBs in response to different biological and environmental stimuli. We validate SSiNGLe using two very distinct sequencing techniques and apply it to derive global profiles of SSBs in different biological states. Strikingly, we show that patterns of SSBs in the genome are non-random, specific to different biological states, enriched in regulatory elements, exons, introns, specific types of repeats and exhibit differential preference for the template strand between exons and introns. Furthermore, we show that breaks likely contribute to naturally occurring sequence variants. Finally, we demonstrate strong links between SSB patterns and age. Overall, SSiNGLe provides access to unexplored realms of cellular biology, not obtainable with current approaches.

## Abstract (web summary)

Single strand breaks represent the most common form of DNA damage yet no methods to map them in a genome-wide fashion at single nucleotide resolution exist. Here the authors develop such a method and apply to uncover patterns of single-strand DNA “breakome” in different biological conditions.

## Introduction

DNA damage is now widely recognized as a major reason behind cancer and many other aging-associated diseases and as such represents a very important issue for human health^1,2^. While multiple types of DNA lesions exist, SSBs are considered the most common type of DNA damage^3^. These lesions can represent sites of oxygen radical DNA damage, intermediates in excision DNA repair pathway and products of unresolved intermediates of enzymes such as topoisomerases^3^. SSBs can further deteriorate into highly toxic double-strand breaks (DSBs) by stalling or collapsing replication forks^4^. However, by themselves SSBs can also represent a major issue for cells as they can inhibit progression of RNA polymerase^5^ and in some cases cause apoptosis^6,7^. The importance of this type of lesion is underscored by existence of dedicated cellular pathways that deal with every step of fixing SSBs from detection to processing to repair^3^. Defects in these pathways can lead to cellular sensitivity to genotoxic stress, embryonic lethality and a number of neurodegenerative diseases^8^.

The remarkable progress in appreciation of the fine details of the SSB repair machinery, however, stands in stark contrast with total absence of methods to map endogenous SSBs in a global, unbiased and genome-wide fashion with nucleotide precision. This gap in available methodologies also contrasts with a suite of comprehensive approaches developed for mapping DSBs with nucleotide-level resolution, such as BLESS^9^, BLISS^10^ and others (reviewed in ref. ^11^). To the best of our knowledge, only one SSB genome-wide mapping method can provide comprehensive and unbiased data^12^. The procedure relies on the 3′OH group of an SSB to prime a DNA polymerase I nick-translation reaction that labels downstream DNA with a biotinylated nucleotide^12^. The labeled DNA is then purified and subjected to next-generation sequencing (NGS)^12^. However, this approach maps a region of DNA, quite possibly extending thousands of bases from the original SSB, thus precluding identification of the lesion with nucleotide precision. On the other hand, a nucleotide-level method to map sites of excision repair has been developed^13^; however, it cannot provide information on breaks generated by other mechanisms.

Thus, all this leads to a total dearth of knowledge of nucleotide-level genome-wide patterns for this critical type of DNA lesion. Here, we develop and validate an approach, SSiNGLe (single-strand break mapping at nucleotide genome level) that can provide nucleotide-level maps of native SSBs genome-wide. We implement this approach to work with two NGS platforms: (1) a 3rd generation single molecule sequencing (SMS) platform (Helicos/SeqLL) whose unique capabilities obviate the need for lengthy sample preparation and PCR amplification, and (2) the more commonly used Illumina platform. We show highly consistent and striking patterns of SSBs using both sequencing platforms. Furthermore, the results show that the genomic pattern of breaks—the SSB “breakome”—has strong potential to represent a novel dimension describing state of a biological system and a novel source of blood-based biomarkers, with potentially yet undiscovered connections to aging.

## Results

### Overview and validation of SSiNGLe-SMS and SSiNGLe-ILM

**Fig. 1:**
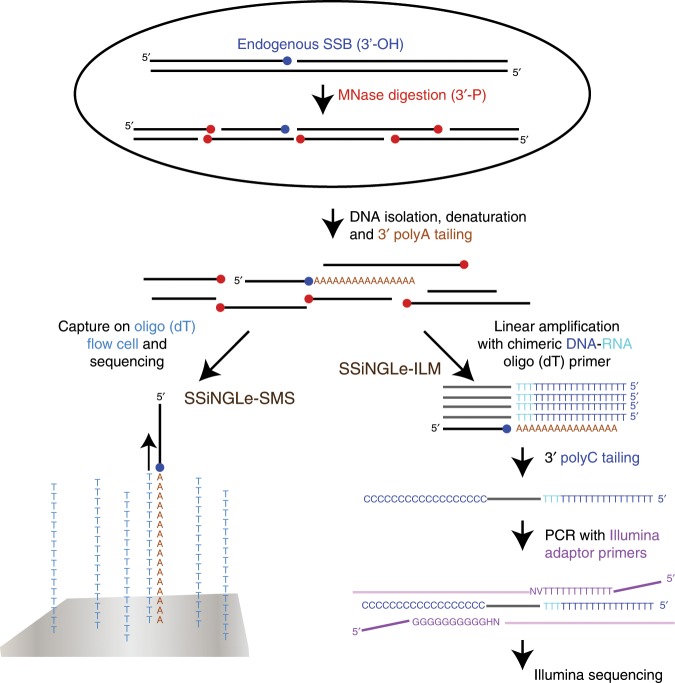
Overview of SSiNGLe-SMS and SSiNGLe-ILM. DNA with 3′-OH SSB (blue circle) is fragmented in situ with MNase that leaves 3′-P termini (red circles) that cannot be tagged with the polyA using TdT. After tailing, the native DNA is either sequenced directly on oligo(dT) flow cells (SSiNGLe-SMS) or put through the additional depicted steps for SSiNGLe-ILM.

**Fig. 2:**
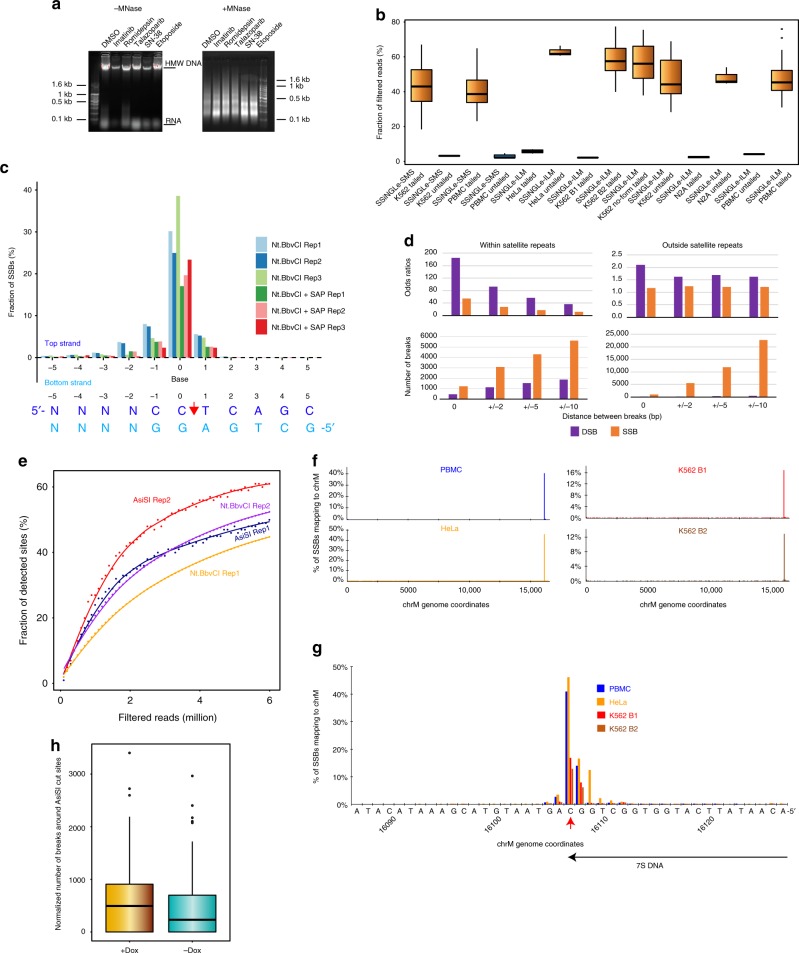
Validation of SSiNGLe-SMS and SSiNGLe-ILM. **a** Agarose gel electrophoresis analysis of genomic DNA isolated from cells treated for 48 h with indicated drugs and then digested (right) or not digested (left) with MNase. Signal at the bottom of the gel with un-digested material represents residual degraded RNA. **b** Fraction of uniquely aligned reads that survives filtration for the adjacent endogenous polyA stretches in different tailed and untailed samples. **c** Results of three replicas (“Rep 1” – “Rep 3”) of in situ digestion of nuclei from H_2_O_2_-treated K562 cells using Nt.BbvCI with or without SAP. Fraction of SSBs (*Y*-axis) found by SSiNGLe-ILM (relative to total SSBs detected in each sample) mapping to each of the indicated bases of the Nt.BbvCI sites and flanking sequences (*X*-axis) for the top and bottom strands of the site. The cleavage site is shown with the red arrow. **d** Overlap between SSBs or DSB found by SSiNGLe-ILM in HeLa cells with DSBs found by the BLESS protocol^9^. The odds ratios and numbers of overlapping breaks are shown for the different distances between the breaks in the two datasets. **e** Fraction of detected AsiSI and Nt.BbvCI sites (*Y*-axis) at different numbers of filtered reads (*X*-axis). Each experiment was done in two replicas. **f** Median fraction of SSBs (*Y*-axes) mapping to each base (*X*-axes) of the minus or H strand of the chrM genome is shown for each of the three sample types. **g** Zoom-in on the region of the major peak in the panel (**f**). The red arrow represents the previously reported 3′ end of 7S DNA^21,22^. Only background-level signal can be observed on the opposite strand (Supplementary Table 3). **h** Box plots of normalized scores of SSBs at each position around AsiSI cut sites (±10 bp) for 28 samples (14+Dox and 14 −Dox). Normalized score = $\frac{{{\rm{SSBs}}\;{\rm{detected}}\;{\rm{at}}\;{\rm{each}}\;{\rm{position}} \ast 10^9}}{{{\rm{Total}}\;{\rm{number}}\;{\rm{of}}\;{\rm{SSBs}}}}$.

The essence of our approach is based on tagging a free 3′-OH terminus representing an SSB by addition of the polyA tail using terminal transferase (TdT) (Fig. 1). Prior to the tagging, the high-molecular weight (HMW) DNA is fragmented with micrococcal nuclease (MNase) that leaves 3 PRIMER -phosphate ends^14^ that cannot be used as substrates for tailing by TdT and thus not detectable by our procedure (Fig. 1). This step avoids generating breaks via mechanical shearing of HMW DNA during subsequent purification and reduces its size to a range suitable for downstream NGS analyses. Prior to fragmentation, cells are crosslinked in situ with formaldehyde. Nuclei isolated from the crosslinked cells are then treated with MNase to fragment genomic DNA to a range of 150–500 base pairs (Fig. 2a). Following MNase inactivation, the nuclei are subjected to proteinase K treatment and crosslink reversal. The fragmented genomic DNA is then isolated, denatured and polyA-tailed with TdT. The polyA tags can then be used to capture and identify the positions of SSBs genome-wide using NGS (Fig. 1).

Initially, we tested utility of this method using a 3rd generation NGS platform based on Helicos SMS technology^15^ ideally suited for this task. This platform can directly sequence single-stranded (ss)DNA^16^ possessing 3′ polyA tails on a flow-cell containing millions of oligo(dT) molecules^16^ that can both capture the polyA-tailed molecules and prime sequencing reactions (Fig. 1). Thus, the tailed native genomic DNA can be directly used for SMS without any additional amplification or preparation steps (Fig. 1). We applied this strategy (named SSiNGLe-SMS) to profile SSBs in human leukemia K562 cells treated with different anti-cancer medications (see Methods) and PBMC’s isolated from 40 donors. After filtering aligned reads for proximity to internal polyA stretches in the genome, 2–4% of reads remained in the un-tailed controls compared to 20–60+% in the tailed samples with median 42.6% in both sample types (Fig. 2b, Supplementary Data 1). Furthermore, the number of the post-filtered reads in the untailed controls corresponded to only 0.2–3.4% of median number of post-filtered reads in the tailed samples (Fig. 2b, Supplementary Data 1).

Encouraged by these results, we further sought to adapt this approach to a more widely used Illumina NGS platform that also generates longer reads allowing for a more accurate mapping of SSBs. To accomplish this, we added the following three key steps to the SMS protocol (Fig. 1). First, the polyA-tailed molecules were linearly amplified using 10 cycles of primer extension with a chimeric DNA–RNA oligo-d(T)_50_-r(T)_3_ oligonucleotide consisting of 50 2′-deoxythymidine and 3 thymidine nucleotides at the 3′ end. The last 3 RNA residues were included to prevent tailing of the oligonucleotide with TdT at the next step since the enzyme cannot use RNA as a substrate. Second, the products of primer extension were tailed at the 3′ end with polyC tail using TdT. Finally, the desired molecules containing oligo-d(T)_50_-r(T)_3_ sequences at the 5′ ends and polyC-tail at the 3′ ends were exponentially PCR amplified with oligonucleotides containing Illumina adaptor sequences (Fig. 1) and subjected to NGS. As in the SMS experiments, samples without the polyA-tailing served as controls.

We then further tested various performance metrics of the Illumina based approach (SSiNGLe-ILM). First, we tested whether it could indeed detect SSBs by using a nicking site-specific restriction endonuclease Nt.BbvCI that can digest only the top strand of CCTCAGC site. Crosslinked nuclei were digested with Nt.BbvCI prior to MNase fragmentation. Indeed, as shown in Fig. 2c, we observed a very high enrichment (152-fold) of breaks mapping to the correct strand of the CCTCAGC site. Second, digestion with this enzyme allowed to test whether the method indeed has nucleotide-level precision. In fact, about half (43.3–50.7%) of the breaks mapping within ±5 bp of Nt.BbvCI cut sites mapped to the correct base and the vast majority (86.8–94.7%) were within 1 nucleotide of the predicted cut site (Fig. 2c, Supplementary Data 2). Third, we tested whether breaks with termini other than 3′-OH could be detected. To accomplish this, we digested nuclei with Nt.BbvCI in the presence shrimp alkaline phosphatase (SAP) that converts 3′-phosphates to 3′-OH termini. The former termini commonly occur in response to some DNA damaging agents, such hydrogen peroxide treatments, therefore these experiments were done on nuclei of cells treated with H_2_O_2_. Since Nt.BbvCI produces 3′-OH termini, the treatment with SAP would not affect detection of these sites. However, if as expected, additional breaks become detectable after this treatment, then the fraction of Nt.BbvCI reads would decrease in the SAP treated vs un-treated cells and this is exactly what we observed in multiple independent replicas as shown in Fig. 2c and Supplementary Data 2.

Fourth, theoretically, both SSBs and DSBs should be captured by this approach, however since the former outnumber the latter by 2–3 orders of magnitude^3,17,18^, even after treatments with such well-known DSB-inducing drugs as etoposide^19^, the predominant signal is expected to derive from SSBs. Still, we tested ability of our method to detect endogenous DSB’s by two approaches. First, we tested detection of exogenous DSBs caused digestion of fragmented DNA by a well-characterized rare-cutting restriction endonuclease AsiSI^20^ (Methods) represented by 1242 restriction sites or 2484 cleavage sites in the human genome. Indeed, we could detect breaks on both strands of the expected target sites, the majority of which (51.6%) mapped to the correct base and most (84.4%) within 1 base of the expected cleavage site. Second, we tested detection of endogenous DSBs by comparing SSiNGLe-ILM profiles of breaks in HeLa cells (see below) and those obtained using BLESS protocol^9^. We defined DSBs as two SSBs occurring on opposite strands in the SSiNGLe-ILM data. Indeed, we observed a statistically significant overlap between the two datasets, with a very strong reproducible enrichment in satellite repeats (odds ratios 208–343) (Fig. 2d, Supplementary Data 3, Supplementary Note 1). The enrichment was highest with the exact match of genomic coordinates and decreased with increasing the overlap between the breaks in the two datasets (Fig. 2d, Supplementary Data 3, Supplementary Note 1). The overlap was significantly lower, but still statistically significant in the regions outside of the satellite repeats (Fig. 2d, Supplementary Data 3, Supplementary Note 1).

Fifth, we investigated the sensitivity of detection of SSBs and DSBs using SSiNGLe-ILM. Deep sequencing of the above-mentioned samples digested with AsiSI and Nt.BbvCI revealed that 2.8 M uniquely aligned and filtered reads can be sufficient to detect 50% of the 2,484 AsiSI sites in the human genome (Fig. 2e, Supplementary Fig. 1). This number increases to 5.1 M such reads required for detection of 50% of the 1.4 M Nt.BbvCI sites (Fig. 2e). Overall, we estimate that ~50M and ~18M filtered reads would be required to detect close to 100% of the AsiSI and Nt.BbvCI sites. We next examined sensitivity of break detection by diluting human K562 cells with mouse N2A cells in different proportions (1:2, 1:4 and 1:9) and measuring detection of human breaks. Indeed, we could detect breaks in human cells even at lowest 1:9 dilution (Supplementary Table 1).

Finally, we tested the ability of our method to detect endogenous breaks using two approaches. First, the mitochondrial 7S DNA is a naturally occurring small single-strand DNA molecule with 3′OH terminus whose exact position has been previously established^21,22^. Thus, the 3′OH terminus of this molecule can serve as a surrogate for a characterized naturally occurring SSB. Indeed, as shown in Fig. 2f, we observed a clear peak of accumulation of SSBs on the expected (minus or H (heavy)) strand of the mitochondrial genome mapping to the location of the 3′ end of the 7S DNA molecule in PBMC, HeLa, and K562 samples. Strikingly, the exact position of that peak coincided precisely and on the correct strand with the position reported earlier^21,22^ in all three types of samples (Fig. 2g, Supplementary Tables 2, 3). The fraction of mitochondrial reads mapping to the 3′ end of 7S DNA was reproducible between the two biological replicas of K562 cells yet varying among the three sample types (Fig. 2g), likely reflecting reported differences in abundances of the molecule among different cell types^23^. Overall, these results show that our method can consistently and accurately detect endogenous SSBs with nucleotide-level resolution. Second, we generated stable K562 cell line expressing doxycycline (Dox) inducible nuclear-targeted AsiSI endonuclease. Comparison of multiple +Dox vs −Dox samples revealed 1.27-fold enrichment of breaks within ±10 bp window from the expected cut sites the +Dox samples with *p*-value 0.008 (Wilcoxon signed-rank test) (Fig. 2h, Supplementary Data 4, Supplementary Note 1) suggesting that our method can indeed detect rare endogenous breaks.

We then applied SSiNGLe-ILM to profile breaks occurring in four sample types. The first sample type was human leukemia K562 cells treated with seven anti-cancer medications—etoposide (inhibitor of Topoisomerase II), SN-38 (inhibitor of Topoisomerase I), romidepsin (inhibitor of histone deacetylases), imatinib (inhibitor of BCR-ABL oncogene present in K562), talazoparib (PARP inhibitor), 10074-G5 (c-Myc inhibitor), and YM-155 (DNA intercalator)—and DMSO control for 6, 12, 24, 36, and 48 h in two independent biological replicas. One of the major purposes of this sample type was to study profiles of SSBs happening during early stages of apoptotic DNA fragmentation characterized by production of HMW 50–300 kb DNA pieces^24,25^. While genomic landscape of DSBs occurring at the late stage when DNA gets fragmented into 200 bp oligonucleosomal fragments^26^ has been characterized^27^, the landscape of breaks happening at the first HMW stage remains unknown. In fact, previous studies suggested prominent role of SSBs occurring during the latter^28,29^. After 36–48 h, all of the treatments including DMSO caused marked increase in apoptotic cells (Supplementary Fig. 2, Supplementary Table 4), while most of the DNA was still in HMW (Fig. 2a) thus allowing for investigating break pattern during the early apoptotic stage.

The other two sample types were human HeLa and mouse Neuro2A cell lines with three biological replicas each. Finally, human PBMCs isolated from 84 female donors aged 20–89 years allowed to investigate the break profiles in normal human cells and correlate them with chronological age (see below). Importantly, for all four sample types, similar to the SMS results, 28–77.2% (median 49.9%) of the uniquely mapped Illumina reads from the polyA-tailed samples could survive the filtration for proximity to internal polyA stretches compared to only 2–7% (median 2.8%) in the corresponding un-tailed controls (Fig. 2b, Supplementary Data 1). While broadly used in many methodologies, including DNA break mapping^9^, formaldehyde crosslinking has been reported to cause artifacts^30^ and even DNA damage^31^. To evaluate potential effects of the crosslinking step per se to the breaks detected by SSiNGLe-ILM, we have processed a separate biological replica of K562 cells treated with etoposide, romidepsin and DMSO for 6, 12, 24, 36, and 48 h without the formaldehyde crosslinking step. In these samples, we have observed similar fraction of reads surviving the filtration step as in the crosslinked samples (Fig. 2b). Altogether, the data shown above strongly argue that SSiNGLe-ILM can reproducibly detect bona fide SSBs in different cell types and different species with nucleotide resolution. Therefore, we adopted this protocol for all analyses shown below. However, since it relies on PCR amplification that has the potential to distort the population of nucleic acids, we have profiled breaks from some of the same samples using SSiNGLe-SMS (Methods) to compare the results obtained by the two approaches. For the SSiNGLe-SMS though, for most analyses, we had to combine breaks found in the different samples due to limited number of reads (Supplementary Data 1). However, the higher read counts obtained by SSiNGLe-ILM (Supplementary Data 1) allowed us to investigate the genome profile of SSBs for each drug and each time point individually for all analyses shown below.

### Genomic landscape of SSBs

Since the BLESS approach found enrichment of DSBs in satellite repeats^9^ and we found the highest overlap between DSBs detected by our approach and BLESS on these regions, we first tested for enrichment of breaks detected by SSiNGLe-ILM or SSiNGLe-SMS in various types of repeats. Indeed, we observed the highest enrichment of breaks in satellite repeats in both methods (median odds ratio 5.32, *p*-value < 2.2E–16, binomial test), significantly higher than the second most enriched class—Alu repeats (median odds ratio 1.29, *p*-value < 2.2E–16, binomial test) (Supplementary Data 5). For all analyses described below, we will use breaks that map to non-repetitive portions of the genome unless stated otherwise.

**Fig. 3:**
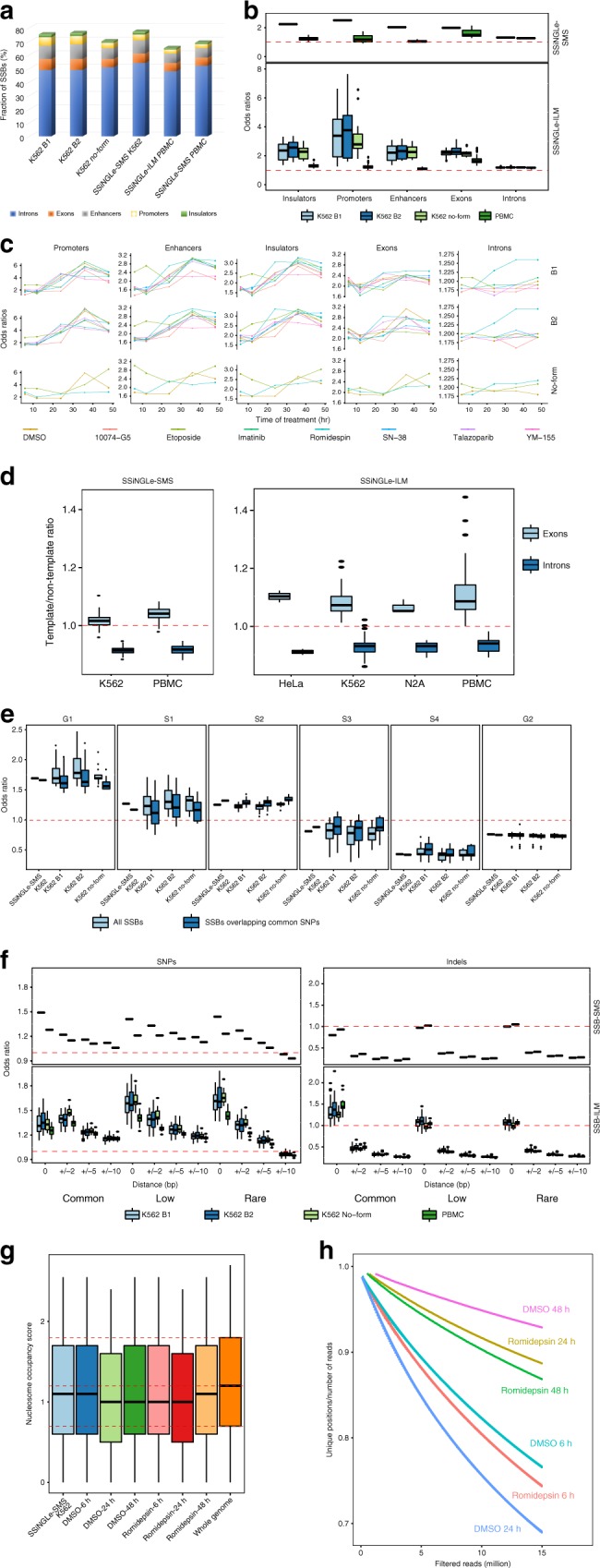
Genomic landscape of SSBs. **a** Average fraction of SSBs falling within each of the indicated genomic elements for different biological replicas of K562 drug treatments (B1 or B2), no-formaldehyde crosslinking (“no-form”), PBMCs and detected using either SSiNGLe-SMS (combined K562 or PBMC data) or SSiNGLe-ILM. **b** Box plots of distributions of odds ratios of overlap between different genomic elements and different sample types. The top panel shows results from SSiNGLe-SMS for K562 (left box plot) and PBMCs (right box plot). The bottom one presents the results from SSiNGLe-ILM with four box plots per element representing K562 B1, K562 B2, K562 no-formaldehyde and PBMCs (left to right). **c** Distribution of odds ratios of overlap between different genomic elements (*Y*-axes) and drug treatment times (*X*-axes) for two biological replicas (B1 and B2) and no-formaldehyde crosslinking K562 samples (SSiNGLe-ILM). **d** Box plots of distributions of template/non-template ratios for exons (left box plot) and introns (right box plot) for different sample types. SSiNGLe-SMS (left panel) and SSiNGLe-ILM (right panel). **e** Box plots of distributions of enrichment of SSBs for the indicated sample types in different phases of the cell cycle (G1, S1–S4 and G2). For each phase and sample types, data from all SSBs (left box plot) and those overlapping common SNPs (right box plot) are shown. The results were obtained with SSiNGLe-ILM. **f** Box plots of distribution of odds ratios of overlap between SSBs and SNPs or indels (common, low frequency and rare) for the indicated distance windows. Top and bottom panels represent respectively SSiNGLe-SMS and SSiNGLe-ILM. For each variant type and distance combination, the top panel shows results from SSiNGLe-SMS for K562 (left box plot) and PBMCs (right box plot), and the bottom panel shows the results from SSiNGLe-ILM for K562 B1, K562 B2, K562 no-formaldehyde and PBMCs (left to right). **g** Box plots of distribution of nucleosome occupancy scores for the indicated sample types and whole genome (SSiNGLe-ILM). **h** Decrease of the complexity of SSBs (unique breaks/ total filtered reads, *Y*-axis) vs increase in the total number of filtered reads (*X*-axis) in the deep sequencing samples from indicated treatments (SSiNGLe-ILM).

Availability of maps of various regulatory elements (promoters, insulators, enhancers, replication timing, various histone modifications, and others) generated by the ENCODE consortium^32,33^ for the K562 cell line used in this study allowed us to evaluate global features of the genomic landscape of SSBs during the time course of drug treatments. The majority of SSBs (65–77%) could be mapped to genes or the regulatory elements (promoters, enhancers and insulators) using either SSiNGLe-ILM or SSiNGLe-SMS (Fig. 3a). SSBs had statistically significant overlap with each of the three types of regulatory regions in each drug and DMSO treatment (Fig. 3b, Supplementary Data 6). Overlap between SSBs and promoters had the highest significance as judged by both odds ratios and *p*-values as shown by SSiNGLe-ILM in individual samples (Fig. 3b, Supplementary Data 6) and SSiNGLe-SMS in the combined samples (Supplementary Data 6). Consistent with the enrichment in the regulatory elements, we observed significant overlap with histone marks associated with active chromatin regions and CTCF binding sites (Supplementary Data 7, Supplementary Note 1). Interestingly, Gene Ontology (GO) analysis of genes with breaks in promoters revealed clear and consistent enrichment of terms associated with RNA processing, cell cycle control, and DNA repair (Supplementary Data 8, 9, Supplementary Note 1, Supplementary Fig. 4).

Surprisingly, analysis of the odds ratios of the enrichment in the individual samples demonstrated a rather interesting and a highly consistent pattern for each regulatory element. Specifically, the odds ratios for each regulatory element increased with the timing of drug treatment to peak at 24 or 36 h coinciding with the increase in the fraction of apoptotic cells and then decreased at 48 h while remaining significant throughout the entire time course (Fig. 3c, Supplementary Data 6). Overall, the odds ratios for promoters, enhancers and insulators varied in the corresponding ranges of (1.3–7.6), (1.4–3.2), and (1.3–3.4) (Supplementary Data 6). Since all treatments, including the DMSO control, exhibited time-dependent increase in apoptosis usually observable at 24 h and peaking at the late 36–48 h time points (Supplementary Fig. 2, Supplementary Table 4), it is highly likely that the time-dependent enrichment pattern reflected different phases of apoptosis shared by all treatments (also see below). Interestingly, the enrichment of the above GO terms was also most pronounced at the 24–48 h time points (Supplementary Data 8, Supplementary Note 1, Supplementary Fig. 4).

DNA breaks found by either SSiNGLe-SMS or SSiNGLe-ILM also had significant tendency to map to exons and introns with SSiNGLe-ILM odds ratios of 1.65–3.14 for the former and 1.16–1.27 for the latter (Fig. 3b, Supplementary Data 10). The exonic odds ratios for individual samples by SSiNGLe-ILM also increased with the timing of drug treatment as found in the regulatory regions, while those with introns did not with exception of romidepsin (see below) (Fig. 3c, Supplementary Data 10). Surprisingly, the genes with breaks in exons were significantly enriched in neuronal functions (Supplementary Data 11–13, Supplementary Note 1, Supplementary Fig. 4).

Template strand-asymmetry in transcribed regions is a well-known theme in DNA damage and DNA repair^11^. Therefore, we investigated the distribution of breaks on template and non-template strands of exons and introns of annotated human genes. For each sample, we calculated the numbers of breaks mapping to template and non-template strands separately of all annotated exons and introns and then calculated corresponding template/non-template ratios. Surprisingly, the ratios were consistently higher for exons than introns with both techniques and both biological replicas tested with SSiNGLe-ILM. With SSiNGLe-ILM, with the exon template/no-template ratios ranged from 1.01 to 1.22 and the intron one 0.86–1.02 (Fig. 3d, Supplementary Data 14). With SSiNGLe-SMS, with the exon template/no-template ratios ranged from 0.96 to 1.104 and the intron one from 0.884 to 0.947 (Fig. 3d, Supplementary Data 14). These differences of template/non-template ratios between exons and introns were statistically significant across all K562 samples and for both methods: *p*-value < 2.2E–16 (SSiNGLe-ILM) and 1.53E–9 (SSiNGLe-SMS) (Wilcox Rank Sum Test), suggesting that relatively more breaks occur on the template strands of exons than introns. Furthermore, the ratios exhibited remarkable consistency across all K562 samples with corresponding coefficients of variations for exons and introns being 0.038 and 0.031 (SSiNGLe-ILM) and 0.029 and 0.017 (SSiNGLe-SMS) (Fig. 3d, Supplementary Data 14). To further validate this phenomenon, we calculated the same ratios for the exogenous nicks introduced by the Nt.BbvCI enzyme in either exonic or intronic regions in the two replicas of the deep-seq data. The observed vs expected ratios for nicks in Nt.BbvCI sites located in exons (1.03 and 1.04) were quite similar to those found in introns (1.02 and 1.03). However, the template/non-template ratios outside of the cut sites in these samples for exons (1.02 and 1.05) were more different than those for introns (0.97 and 0.96). These results suggest that the observed differences between the strands are not introduced by the procedure itself, but rather reflect a bona fide endogenous phenomenon.

To assess whether the breaks correlated with replication timing, we took advantage of the Repli-seq dataset for the K562 cell line across six cell cycle fractions^34^. We calculated replication ratios describing enrichment (>1) or depletion (<1) of breaks relative to the whole genome background for each cell cycle fraction and each sample (Fig. 3e, Supplementary Data 15, Methods). We found strong enrichment of the breaks in regions replicating in the early G1/G1b, S1 and S2 phases with median replication ratios for all K562 samples of 1.75, 1.27, and 1.22 and depletion of breaks regions replicating in the late S3, S4, and G2 phases with the corresponding median ratios of 0.81, 0.43, and 0.75 (Fig. 3e, Supplementary Data 15). Moreover, we saw a general tendency for the breaks found in the later stages of the drug treatments to occur in earlier replicating regions compared to the breaks found at the early stages of the drug treatments (Fig. 3e, Supplementary Data 15).

DNA damage could potentially result in mutations or DNA rearrangements. Therefore, we asked whether SSBs correlated with SNPs and indels found in Phase 3 of the 1000 Genomes project^35^. To answer this question, we tested exact overlap of coordinates of the variants and breaks and also extended coordinates of the latter by ±2, 5 or 10 bp. The significance of the overlap with SNPs as measured by the odds ratio was the highest for the exact overlap (low frequency and rare SNPs) or for nearby (±2 bp) (common SNPs). It gradually dropped with extending the boundaries of the breaks to ±5 and ±10 bp, but still remained significant for all but rare SNPs within ±10 bp window (Fig. 3f Supplementary Data 16). Similar trends could also be observed with SSiNGLe-SMS analysis (Fig. 3f, Supplementary Data 16). Interestingly, SSiNGLe-ILM data also revealed significant overlap with indels, but only when exact coordinates of breaks and indels were compared (Fig. 3f, Supplementary Data 17). Extension of the coordinates resulted in sharp drop in the odds ratio (Fig. 3f, Supplementary Data 17). Similar trends were revealed by SSiNGLe-SMS (Fig. 3f, Supplementary Data 17); however, the significance of the overlap with the exact matches was not reached, possibly due to the lower number of reads in this method compared to SSiNGLe-ILM.

Since DNA breaks tend to associate with sequence variants and the latter tend to associate with late replicating regions^36^, we investigated replicating timing of DNA breaks associated with SNPs and indels. Indeed, we observed a shift towards late replication for DNA breaks found within 2 bp of common SNPs (Fig. 3e). Thus, while overall DNA breaks tend to occur in early replicating regions, those associated with SNPs tend to replicate later in the cell cycle. Interestingly, when normalized for the occurrence of the variants, SNP-associated breaks were significantly (2–3 fold) depleted from exons and the regulatory elements compared to all breaks (Supplementary Data 18). On the other hand, the SNP-associated breaks were significantly enriched in histone marks associated with silent chromatin, particularly in H3K9me3 (4 + fold) (Supplementary Data 18). Finally, as expected from association with sequence variants, genomic positions corresponding to DNA breaks do have less evolutionary conservation than neighboring sequences (Supplementary Note 1, Supplementary Table 5).

Topoisomerase cleavage generates transient breaks in order to relax or untangle genomic DNA. While these breaks possess termini other than 3′OH, they are converted into such during the DNA repair and thus in principle should be detectable using our approach. Indeed, we observed a statistically significant enrichment of our breaks in cleavage cluster regions for the topoisomerase Top2A previously found in K562 cell line^37^: odds ratios 1.45–1.64 SSiNGLe-ILM (*p*-values < 2.2E–16, binomial test) and odds ratio of 1.67 using SSiNGLe-SMS (*p*-value < 2.2E–16, binomial test). However, only 2.4–2.7% of all breaks detected by the SSiNGLe-ILM protocol could be mapped to these sites arguing that majority of breaks are generated using processes other than topoisomerase cleavage or at least, not by the Top2A enzyme (Supplementary Data 19).

MNase treatment would be expected to preferentially target the nucleosome-free regions of DNA, this potentially depleting breaks occurring at those regions. To investigate this possibility, we took advantage of nucleosome positioning data obtained by sequencing of MNase-digested K562 DNA^38^. We calculated distribution of nucleosome occupancy scores for breaks and compared it with the whole genome distribution. Overall, the breaks detected by either SSiNGLe-SMS or SSiNGLe-ILM had a slight but statistically significant (*p*-value < 2.2E–16 for SSiNGLe-SMS and SSiNGLe-ILM) tendency to occur in regions of lower nucleosome occupancy (Fig. 3g). These results suggest that (1) the pattern of breaks is different from the pattern of the whole genome and (2) the procedure does not appear to bias against the regions of lower nucleosome occupancy.

We explored the landscape of breaks by sequencing six samples (romidepsin and DMSO treatments, 6, 24, and 48 h) at higher depths (Supplementary Note 1). The ratio of unique breaks to total number of filtered reads indicative of “breakome” complexity decreased with the increase in sequencing depth as expected (Fig. 3h). Interestingly, majority of the observed signal (98–99%) did not overlap on both strands within ±2 bp, suggesting that as expected, the vast majority of the signal was contributed by SSBs. Strikingly, hotspots of SSBs represented ≥4 reads had stronger overlaps with multiple genomic features described above compared to singleton SSBs (Supplementary Note 1, Supplementary Fig. 3).

Remarkably, a number of key genomic patterns described above for K562 were also found in normal human PBMCs (Supplementary Note 1). Furthermore, we observed the same trends in the samples without the formaldehyde crosslinking step (Fig. 3, Supplementary Data 1, 5–7, 10, 14–17, 19, Supplementary Note 1) arguing that the observed results are not artifacts of the crosslinking.

### SSBs “breakome” provides a novel biological dimension

We then asked whether a global profile of SSBs—the “breakome”—can correlate with a biological state. A positive answer to this question has two implications. First, technical—artificial breaks generated by the procedure itself would not be expected to correlate with a specific biological state. Therefore, clustering of samples according to their biological states would suggest that artificial breaks represent the minority of the data. Second, biological—non-random association of breaks with biological states would suggest potential biological significance of the former.

**Fig. 4:**
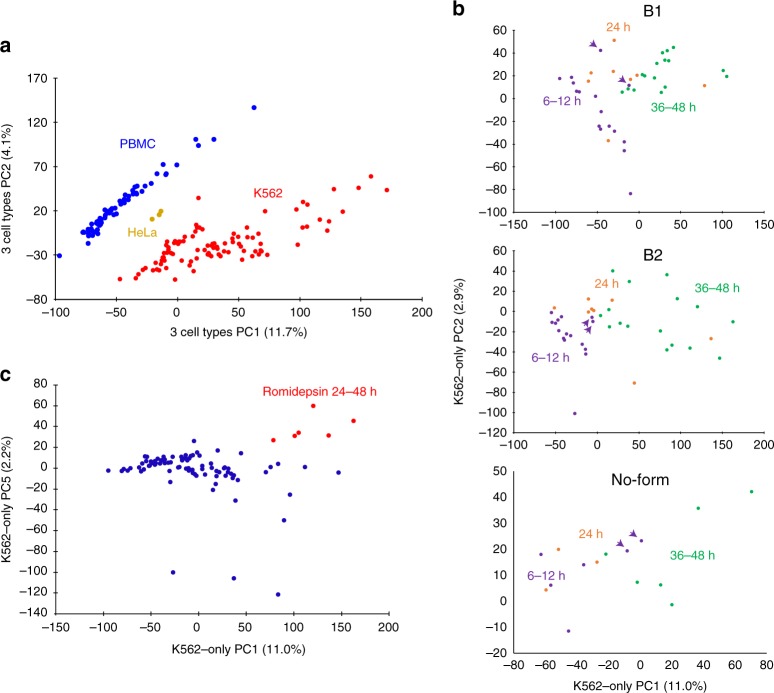
Association of SSB “breakome” with biological states. Results of PCA analyses based on profiles of breaks in genic regions detected using SSiNGLe-ILM. **a** clustering of three cell types—95 K562 samples (two biological replicas and no-formaldehyde samples), HeLa (three biological replicas) and 66 PBMC samples. **b** Separation of early (6–12 h), middle (24 h) and late (36–48) time points of treatments in two biological replicas (B1 and B2) and no-formaldehyde K562 samples. Purple arrowheads indicate early time points of etoposide treatments. **c** Clustering of middle-late time points of K562 romidepsin treatments. PCA was performed on all three cell types (**a**) or just the K562 samples (**b**, **c**).

We generated density of DNA breaks detected with SSiNGLe-ILM for every strand of every known human gene and used these values to cluster all 164 samples (95 drug-treated K562, 66 PBMC’s and 3 HeLa samples) using principal component analysis (PCA). As shown in Fig. 4a, different cell types (K562, PBMCs, and HeLa) could be clearly separated on the basis of this analysis. We then asked whether “breakome” can provide a more precise molecular signature that could distinguish different molecular states within a cell type by performing additional PCA analysis, using only the 95 drug-treated K562 samples (Fig. 4b, c). The most significant PC1 and PC2 dimensions could consistently separate the samples according to the time of treatment (Fig. 4b). The samples in the early (6–12 h), middle (24 h) and late (36–48 h) time points could also be separated in the two different biological replicas as well as in the no-formaldehyde samples (Fig. 4b). The latter provides additional evidence that the formaldehyde crosslinking does not contribute a significant fraction of the breaks in our procedure (also see above). Since, as mentioned above, all treatments, including the DMSO control, exhibited time-dependent increase in apoptosis (Supplementary Fig. 2, Supplementary Table 4), it is highly likely that the observed clustering based on the PCA analysis as well as the time-dependent patterns of enrichment in different genomic elements (see above) represented molecular signatures associated with different phases of apoptosis shared by all treatments.

On the other hand, we could also detect signatures specific to particular drugs. For example, early time points (6–12 h) of etoposide treatments consistently clustered with later time points of other drugs (Fig. 4b). Furthermore, 6 h etoposide treatment had higher odds ratios in the regulatory regions and introns (but not exons), compared to all other drugs (Fig. 3c). And, the middle and later (24–48 h) time points of romidepsin treatment clustered separately from other drug treatment samples (Fig. 4c) and had consistently higher enrichments in introns compared to other drugs (Fig. 3c). Thus, the “breakome” molecular signature could not only distinguish between different cell types, but also detect fine differences between different biological states within the same cell type.

### Association between SSB “breakome” and aging

**Fig. 5:**
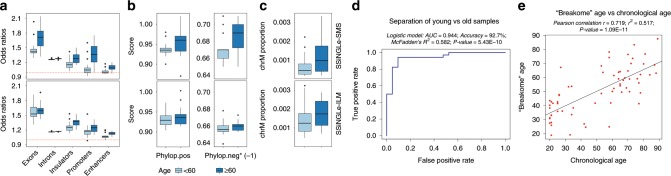
Association of various genomic features of SSBs with age. PBMC samples were stratified into <60 and ≥60 years old groups (respectively 18 and 22 for SSiNGLe-SMS and 23 and 21 for SSiNGLe-ILM). **a** Box plot distributions of the odds ratios of enrichment of SSBs in exons, introns, promoters, insulators and enhancers. **b** Box plot distributions of average PhyloP positive or negative scores (absolute values are shown) in ±20 bp windows. **c** Box plot distributions of the breaks mapping to chrM. Upper panels represent SSiNGLe-SMS while lower panels SSiNGLe-ILM. **d** The ROC curve and properties of the logistic model built to classify 21 “young” (≤30 years-old) and 34 “old” (≥58 years-old) individuals. **e** The scatter plot of “breakome” age (*Y*-axis) vs chronological age (*X*-axis). The “breakome” age was predicted using multiple linear model for the 66 samples of all ages. Samples in (d) and (e) were profiled using SSiNGLe-ILM.

The association between DNA damage and a number of human conditions^1–3^ make genomic patterns of SSBs attractive and previously unexplored tool for development of molecular biomarkers. Considering that DNA breaks have widely implicated in human aging^1^, we investigated potential relationship between various SSBs genomic “breakome” features and chronological age in human blood. We performed this analysis separately using the 84 PBMC samples and splitting them into two different cohorts of 40 and 44 individuals profiled respectively with SSiNGLe-SMS and SSiNGLe-ILM. We found significant association between chronological age and three types of genomic patterns in PBMC SSBs (Fig. 5a–c). First, with age breaks had a higher tendency to occur in genes and functional genomic regions (Fig. 5a). Spearman correlations between the age and odds ratios of enrichment in the 1st cohort using SSiNGLe-SMS for exons, introns, promoters, insulators, and enhancers were respectively: 0.44 (*p*-value 4.3E–3), 0.42 (*p*-value 7.8E–3), 0.51 (*p*-value 7.2E–4), 0.46 (*p*-value 3E–3) and 0.46 (*p*-value 2.8E–3) were encouraging. The same trends were observed for the 2nd cohort using SSiNGLe-ILM with the corresponding correlations of 0.28 (*p*-value 0.06), 0.29 (*p*-value 0.05), 0.5 (*p*-value 5.7E–4), 0.65 (*p*-value 1.5E–6) and 0.68 (*p*-value 4.4E–7) (Fig. 5a).

Second, the age “breakome” correlated with evolutionary conservation of sequences flanking the breaks. For this analysis, we used PhyloP scores that measure both negative selection (positive PhyloP scores) and accelerated evolution (negative PhyloP scores)^39^. For each break, we calculated separately average positive and negative scores for ±20 bp windows around each break and then calculated average scores for each sample. Spearman correlations between the average conservation score and age were 0.32 and −0.42 for positive and negative PhyloP scores (corresponding *p*-values 4.6E–2 and 6.2E–3) using SSiNGLe-SMS (Fig. 5b). The corresponding values for SSiNGLe-ILM were 0.24 and −0.26 (Fig. 5b). Thus, consistent with the age-dependent bias towards the functional elements, in older individuals breaks tend to occur in sequences under higher evolutionary pressure—either sequence conservation (negative selection) or accelerated evolution—and, interestingly, favoring the latter more than the former.

Third, considering the importance of mitochondrial functional in different theories of aging, we investigated whether breaks in the mitochondrial genome (chrM) increased with age. Indeed, we found that the proportion of breaks mapping to chrM became larger with age (Fig. 5c). Spearman correlation of the fraction of chrM breaks with age was 0.37 (*p*-value 2.0E–2) for SSiNGLe-SMS and 0.40 (*p*-value 6.9E–3) for SSiNGLe-ILM (Fig. 5c).

We then ascertained whether we could stratify individuals into “young” and “old” categories based on the age-related PBMC “breakome” features. To do that, we split the 66 PBMC samples profiled using SSiNGLe-ILM into ≤30 (21 individuals) and ≥58 (34 individuals) years old groups and built a logistic regression model on 55 such samples. Indeed, the model could classify the samples with accuracy of 92.7%, AUC value of 0.944, McFadden’s pseudo *R*^2^ of 0.582 and *p*-value 5.43E–10 (Wilcoxon Rank Sum Test) (Fig. 5d). We then examined the nature of this relationship by building a multiple linear regression model, using the same features to predict the “breakome” age of 66 samples (*p*-value 5.12E–08) and compared predictions from that model with the actual chronological age (Fig. 5e). Indeed, the two showed high correlation (Pearson *r* = 0.719, *p*-value 1.09E–11) suggesting that a linear model for diagnosing ‘breakome age’ will be plausible (Fig. 5e).

## Discussion

In this work, we present and extensively validate a method, SSiNGLe, to map native SSBs at nucleotide resolution genome-wide, in a reproducible, species-independent fashion and in multiple cell types and conditions. The method detects breaks with 3′-OH termini, however, in theory the method should detect lesions with different types of 3′ termini after they get converted to 3′-OH ends during DNA repair. Still, some lesions, for example those caused by covalent crosslink between topoisomerases and DNA would not be directly detected by this method. However, breaks with non-3′-OH termini could be un-masked by treating nuclei with enzymes that convert such termini into 3′-OH as shown by the SAP treatment. Overall, theoretically, the patterns of breaks detected by this procedure would likely represent a combination of lesions produced by a variety of mechanisms: breaks directly induced by exogenous factors (i.e., drug treatments) and/or their repair intermediates, products of DNA metabolism, stalled replication forks, apoptotic or sub-apoptotic DNA fragmentation and other mechanisms (see below). Still, the net outcome from all these potential contributing sources of breaks results in non-random genomic patterns of SSBs found in this work with each such pattern potentially revealing novel and tantalizing biological phenomena.

Association with biological state represents one pattern of SSB “breakome”. Different cell types have different genomic patterns of breaks and so do different biological states of the same cell type. For example, cells in early stages of apoptosis caused by different anti-cancer drugs have different patterns of breaks from the non-apoptotic cells. Enrichment of SSBs in different genomics elements, for example regulatory elements, exons, and introns and others represents another pattern. In fact, the enrichment of SSBs in active promoters, also found in a different SSB mapping experiments albeit not at nucleotide-level resolution^12^, is also consistent with the enrichment of SSBs in early replicating DNA since the early replicating regions are known to be enriched in active chromatin marks^40^. The levels of the enrichment can vary as evidenced by significant increase of localization of breaks in promoters and other regulatory elements at later time points of drug treatments coincident with the increase in apoptosis. These observations could indicate a very complex role played by DNA breaks. For example, recently revealed complex interactions between promoter elements and DNA damage, with a number of examples where DNA lesions fulfilled specific functions, such as transcriptional activation^11,41^. Furthermore, association of SSBs with enhancers and insulators reported here could likewise reveal additional level of complex associations between DNA breaks and chromatin organization. On the other hand, the increase in breaks at the regulatory regions at the onset of apoptosis could be caused by the apoptotic DNA fragmentation machinery. Conceivably, the latter might preferentially target functionally important elements as a part of the programmed self-destruct program.

The non-random distribution of functions of genes containing breaks in promoters or exons represents yet another such pattern. The enrichment of specific functions is reproducible in different biological states with different functions being either specific to one biological state or shared by multiple. Furthermore, genes with breaks in promoters vs those in exons exhibit enrichment of different GO terms. For example, the former were most enriched in functions associated with RNA processing, cell cycle control and DNA repair, while the latter were preferentially connected with nervous system function. While the former functions could potentially be explained by the response to drugs or to the DNA fragmentation caused by apoptosis, the physiological importance of the latter as well as the mechanistic reason for the difference are less clear. We speculate that the difference likely reflects the complex interplay of the different physiological processes that give rise to breaks with the differences in the accessibility and the repair rates (see below) in different genomic elements. Overall, function of breaks in regulatory elements as well as exons and introns of genes represents an interesting area for further exploration.

The overlap between SSBs and sequence variants is a very intriguing pattern. Improper repair of DNA breaks can conceivably lead to mutations. Indeed, DNA breaks have been linked to naturally occurring sequence variants as well as de novo mutations. Local increase in SNP density was observed around hotspots of recombination-associated DSBs^42^. Furthermore, breaks induced by sub-apoptotic activation of DNA fragmentation enzymes can in fact increase mutation rate in cancer cells^43^ (reviewed in ref. ^41^). Consistent with these earlier findings, association between DNA breaks and sequence variants in cancerous K562 and normal PBMC cells found in this work, suggests that SSBs could contribute to mutations in cancer cells and natural sequence variation in human population. Interestingly, while DNA breaks have a tendency to occur in early replicating DNA, those that associate with sequence variants tend to appear in later replicating regions consistent with higher mutation rate found in the latter^36^. Furthermore, depletion of breaks overlapping variants in different genomic elements could indicate differential level of DNA break repair for different parts of the genome, similar to the recent report for mismatch repair^44^.

In this respect, the enrichment of neuronal functions in genes containing breaks in their exons is quite interesting given that mutations in SSB repair pathways lead to neurodegenerative diseases^8^. Conceivably, SSBs could cause somatic mutations (and other problems, such as RNA Pol II stalling) in neuronal functions genes and thus contribute to neurodegeneration, which has previously associated with defective SSB repair^8^. In fact, these results are consistent with accumulation of somatic mutations in human neurons with age^45^. Overall, the connection between SSBs and mutations could have strong implications for our understanding of processes that cause DNA sequence changes in human disease and even potentially in evolution and as such warrants further investigations.

The differential preference for the template strands of exons and introns consistently observed among different treatments and cell types is quite surprising. Currently, we are not aware of any mechanism that could explain this difference; however, we could detect this phenomenon using both SSiNGLe-SMS and SSiNGLe-ILM methods further supporting its existence. The observation might indicate either differences in accessibility of the two strands in exons and introns, or differential repair or potentially differential targeting of apoptotic DNA fragmentation machinery. Interestingly, recent report shows differential targeting of mismatch repair machinery to exons compared to introns via a specific chromatin mark on the former^44^, but it remains to be determined whether similar or different mechanism is involved in the strand preference reported here.

Our approach can detect DNA breaks in PBMC and furthermore, demonstrate correlation between patterns of DNA breaks in these cells and a human condition—chronological age. Furthermore, the same trends could be obtained on the same samples using SSiNGLe-SMS and SSiNGLe-ILM. These results demonstrate the potential utility of DNA break profiles as liquid biopsy biomarker and robustness of our DNA break mapping approach in this regard. Despite multiple lines of evidence relating different types of DNA damage^1^, including SSBs^46^, with aging, no relationships between the genomic profiles of DNA breaks with age has been reported. This contrasts with a number of different biomarkers of aging based on whole-genome profiles of transcriptome^47^ and epigenome^48^ as wells as on other types of physiological parameters^49^ reported in the last few years. The relationships between profiles of SSBs and aging reported here represents an area of potential significant interests for two main reasons. First, profiles of DNA breaks can expand the existing biomarkers of aging. In fact, transcriptome and epigenome predictors were shown to correlate with different phenotypes of aging^47^ and thus comprehensive prediction of such very complex biological phenomenon as biological age will undoubtfully require multiple types of biomarkers. Second, association of SSB profiles with age can reveal yet unknown mechanisms participating in aging. The increase in breaks in functional genomic regions and the change in the template strand preference could conceivably be passive indicators of changes in DNA accessibility (due to age-related changes in epigenome, for example), DNA repair performance in different parts of the genome, gene expression or some other processes occurring with age. Additional investigations, however, including profiling of large cohorts of individuals, are required to validate and further understand the novel mechanisms of aging potentially uncovered by these results.

Altogether, these results demonstrate importance of mapping SSBs in multiple different tissues, cell types and conditions. They suggest that like the other -omics sciences, the SSB “breakome” focused on genome-wide patterns of breaks, could potentially reveal novel insights into disease, aging and development and suggest new potential biomarkers and therapeutic targets. Our approach would allow direct comparisons of the “breakome” profiles occurring in normal blood, brain and other tissues in different stages of development, environmental conditions, age and genetic backgrounds and even comparison of such patterns among different species, for example human and other primates or mammals. Overall, analysis of “breakome” using SSiNGLe can open windows into previously un-anticipated areas of DNA break biology so far not achievable with current technologies.

## Methods

### Biological material and reagents

Drug treatments of K562 cells: Human chronic myeloid leukemia cell line K562 was obtained from Cell Bank of Chinese Academy of Sciences. Cells were cultured in RPMI 1640 (Thermo Fisher Scientific) supplemented with 10% (v/v) heat-inactivated fetal bovine serum (Thermo Fisher Scientific) and 1% (v/v) pen-strep (Thermo Fisher Scientific) at 37 °C and 5% CO_2_. For drug treatments, 1–2 million cells were seeded at 1 million cells per ml of medium per well in 24- (1 ml media) or 6-well (2–3 ml) plates. After 16 h, the following chemicals were added separately: 0.1% DMSO, 0.5 mM H_2_O_2_, 1 μM imatinib (Abmole Bioscience Inc, M3241), 0.0462 µM romidepsin (Abmole Bioscience Inc, M2007), 1 μM talazoparib (Abmole Bioscience Inc, M1732), 1 μM SN-38 (Abmole Bioscience Inc, M3016), 40 μM 10074-G5 (Sigma, 475957), 1 μM YM-155 (Abmole Bioscience Inc, M2342) and 100 μM etoposide (Abmole Bioscience Inc, M2326) and incubated for 6, 12, 24, 36, or 48 h. All drugs (with exception of H_2_O_2_) were dissolved in DMSO, concentration of which was kept at 0.1% in all treatments. All treatments were done in two biological replicas for SSiNGLe-ILM. The following treatments were performed on separate batch of cells for SSiNGLe-SMS: DMSO, etoposide, SN-38, romidepsin, imatinib and talazoparib for 6, 12, 24 (with the exception of imatinib), 36 and 48 h. For the Nt.BbvCI experiments, hydrogen peroxide treatment was done only for 6 h and un-treated cells grown under the same conditions were used as controls.

Additional human and mouse cell lines: Mouse neuroblastoma (N2a) and human cervical carcinoma HeLa cell lines were obtained from National Infrastructure of Cell Line Resource. N2a cells were cultured in MEM/EBSS (HyClone), supplemented with 10% (v/v) heat-inactivated fetal bovine serum (Thermo Fisher Scientific) and 1% (v/v) pen-strep (Thermo Fisher Scientific) at 37 °C and 5% CO_2_. HeLa cells were cultured following at the same condition as K562 cells.

Preparation of PBMCs: Two to four milliliters of peripheral blood from healthy female donors aged between 25 and 89 years were collected in EDTA anti-coagulations tubes. The samples were diluted with 3 ml of PBS at room temperature and carefully layered on top of Solarbio Lymphocyte Separation Medium (Solarbio Life Science). The samples were centrifuged for 30 min at 1000 *g* at 25 °C in 5804R centrifuge (Eppendorf) with acceleration and brake settings of 6 and 0, respectively. The PBMCs formed in the interphase were aspirated and washed twice with 10 ml of PBS by centrifugation at 1000 *g* at 25 °C and used immediately for crosslinking as described below. All donors have given informed consent and the experiments were approved by the ethics review board of the Quanzhou 2nd Affiliated Hospital and School of Biomedical Sciences, Huaqiao University.

Apoptosis detection: Apoptosis was detected on fresh K562 cells using Annexin V-FITC/PI Apoptosis Detection Kit (Solarbio Life Science) according to the manufacturer’s instructions. Fixed cells were directly analyzed using CytoFLEX S flow cytometer (Beckman Coulter). The fluorescence was detected with 488 nm excitation and either 620 (PI) or 525 filter (Annexin V). The data were analyzed with CytExpert 2.0 software.

Generation and analysis of stable K562 cell line expressing inducible AsiSI: Aminoacid sequence of AsiSI protein (http://www.uniprot.org/uniprot/Q83XX1) was codon optimized for expression in human cells, fused with N-terminal SV40 nuclear localization sequence (NLS) and C-terminal nucleoplasmin NLS, and assembled in a lentiviral expression vector under control of the Dox-inducible TRE promoter. Lentivirus was produced by transfecting 293FT packaging cell line. After the lentivirus transfection, 1 million K562 cells stably expressing EYFP also encoded by the lentivirus vector were selected by flow cytometry (BD CytoFLEX) and expanded. Generation of the cell line was outsourced to SyngenTech (Beijing, China). Induction of the AsiSI mRNA following Dox treatments relative to the −Dox controls was on average ~50 fold as detected by RT-qPCR.

Five million AsiSI-expressing K562 cells were grown to 0.5 million cells per ml and subjected to each of the following drug treatment strategies. (1) Dox-only treatment: 1 μg/ml Dox (Macklin Inc, 24390-14-5) was added for 3, 4, or 13 days. (2) Decitabine/DMSO + Dox: 0.1% DMSO or 0.1% DMSO plus 5 μM decitabine (Abmole Bioscience Inc, M2052) was added for 6 days after which 1 μg/ml Dox was added for additional 2, 3, or 6 days. (3) Kinase inhibitors + Dox: 7 μM AZ-20 (Abmole Bioscience Inc, M2412), 17 μM KU-60019 (Abmole Bioscience Inc, M1998), 5 μM NU7441 (Abmole Bioscience Inc, M1809) and 1 μg/ml Dox were added for 2, 4, or 6 days. (4) Decitabine/DMSO + kinase inhibitors +Dox: 0.1% DMSO or 0.1% DMSO plus 5 μM decitabine was added for 8 days after which a mixture of 7 μM AZ-20, 17 μM KU-60019, 5 μM NU7441 and 1 μg/ml Dox was added for additional 2 or 5 days. For each treatment regime, the corresponding −Dox control (Dox substituted with water) was also used.

### SSB detection—steps common to SSiNGLe-SMS and SSiNGLe-ILM

Preparation of nuclei prior to MNase fragmentation: 1–3 million K562, N2a or HeLa cells or 4–6 million PBMCs were crosslinked in 1–2 ml of either the growth medium (K562) or PBS (PBMCs) supplemented with 1% formaldehyde for 10 min at room temperature followed by addition of glycine (Thermo Fisher Scientific) to the final concentration of 1.375 M to quench crosslinking for 5 min at room temperature. The cells were collected by centrifugation at 1500 *g* at 4 °C, and then washed with ice-cold 1X PBS. To prepare nuclei, the crosslinked cells were lysed in a buffer containing 5 mM PIPES (pH8), 3 mM KCL, 0.5% NP-40 (Amresco) for 10 min on ice; followed by centrifugation at 1000 *g* for 10 min at 4 °C. After removal of supernatant, the nuclei were resuspended in 500 µl cold 1X MNase buffer (NEB) and sedimented again under the same conditions. The wash was repeated again.

(Optional) SDS permeabilization: relatively small MNase (17 kDa) can diffuse into nuclei via nuclear pores. However, treatment of DNA with larger enzymes, for example restrictases or SAP, requires permeabilization with SDS. After removal of supernatant, the nuclei were resuspended in 100 µl cold 1X MNase buffer per 1 million nuclei and split into aliquots of 1 million. Each aliquot was incubated with 0.3% of sodium dodecyl sulfate (SDS) for 1 h at 37 °C with gentle mixing. Then, triton X-100 was added to concentration of 1.8% and incubated for 5 min at room temperature. If to be used directly for the MNase treatment, the nuclei were collected by centrifugation at 3500*g* at 27 °C for 10 min and then washed twice with 1X MNase buffer. If subjected to the restriction enzyme digestion, then processed as below. Since K562 nuclei were used in restriction enzyme digestion experiments to validate SSiNGLe-ILM, we processed all K562 and other cell line samples (HeLa and N2a) with the SDS permeabilization step in the SSiNGLe-ILM protocol. However, PBMCs were processed without it. All samples tested with the SSiNGLe-SMS protocol were processed without the SDS permeabilization step.

(Optional) restriction enzyme digestion inside nuclei: the permeabilized nuclei were collected by centrifugation at 3500 *g* at 27 °C for 10 min and then washed twice with a 1X NEB buffer 2.1 (50 mM NaCl, 10 mM Tris-HCl, and 10 mM MgCl_2_). After removal of supernatant, the nuclei were resuspended in 50 µl of 1X NEB buffer 2.1 and treated with 50U of Nt.BbvCI with or without 1U of SAP (NEB) for 2 h at 37 °C, and then for 20 min at 70 °C to inactivate the enzymes. The nuclei were collected by centrifugation at 3500 *g* at 27 °C for 10 min and then washed twice with 1X MNase buffer. Unlike Nt.BbvCI, however, we could not detect DNA cleavage at the AsiSI sites after treating crosslinked nuclei with this enzyme using either real-time PCR on selected sites or our approach genome-wide (data not shown). Therefore, we digested purified DNA after the MNase fragmentation step (below) with AsiSI before tailing and then subjected it to SSiNGLe-ILM procedure.

MNase fragmentation and DNA purification: after removal of supernatant, the nuclei were resuspended in 50 µl of cold 1X MNase buffer with 100 μg/ml BSA per 1 million nuclei and split into aliquots of 1 million. Each aliquot was digested separately with 1200–3000 units of MNase (NEB) and 200 units of RNAIf (NEB) for 30 min on ice. The digestion products were checked on 1% agarose gel after DNA purification to ensure that the majority of DNA was in the range of 150–500 bp as exemplified in Fig. 2a. After MNase digestion, 5.6 µl of 0.5 M EDTA and 150 µl of nuclei lysis buffer (10 mM EDTA, 1% SDS, 10 mM Tris-HCl pH 8) was added to the mix. After 5 min incubation at room temperature, 1 µl of 20 μg/ml proteinase K (Roche) was added followed by incubation for 45 min at 55 °C and 45 min at 65 °C. The DNA was then precipitated with 0.3 M KCl and cold isopropanol for 30 min at −80 °C, followed by 15-min centrifugation at 4 °C at 12,000 *g*, washing with cold 70% ethanol and vacuum-drying. The DNA pellet was then dissolved in 40 μl water followed by purification with 2X volumes of VAHTS DNA Clean Beads (Vazyme). The concentration of DNA was measured using Qubit 3.0 fluorometer and “dsDNA HS Assay” kit (Thermo Fisher Scientific). In parallel, for every sample we isolated DNA using the same procedure but without the MNase treatment step to ensure that the original DNA was present in HMW form as exemplified in Fig. 2a.

Tagging of breaks using polyA-tailing with TdT: 100 ng of the purified fragmented DNA was denatured at 95 °C for 5 min in 19 µl volume containing 2 µl of 10X TdT buffer, 2 µl of 2.5 mM CoCl_2_ and water followed by rapid snap-cooling on ice. TdT (5 units; NEB) and 2 µl of 1 mM (SSiNGLe-SMS profiling of K562 cells) or 10 mM (all other experiments, SSiNGLe-SMS or SSiNGLe-ILM) dATP were then added to denatured DNA to the total volume of 22 µl and incubated at 37 °C for 30 min. To block free 3′-OH ends, 2 µl of 1 mM (SSiNGLe-SMS profiling of K562 cells) or 10 mM (all other experiments, SSiNGLe-SMS or SSiNGLe-ILM) ddNTP (either one of ddCTP, ddGTP, ddATP, or ddTTP) was added to the tailing mix and incubated at 37 °C for additional 30 min, followed by incubation at 70 °C for 10 min to inactivate TdT and either used directly for SMS or processed further for SSiNGLe-ILM.

SMS: 2.5 µl (K562) or 10 µl (PBMCs) of the tailing mix containing respectively 10.4 or 45.5 ng of genomic DNA was directly used for the SMS outsourced to the SeqLL, LLC facility (Woburn, MA, USA) as previously described^50^. For the un-tailed controls, 15 ng (K562) or 65.5 ng (PBMC) of MNase fragmented DNA was sequenced directly without the tailing/blocking steps.

### SSB detection—steps specific to SSiNGLe-ILM

Linear amplification: the tailing mix was purified with 2X volumes of VAHTS beads, eluted in 16.2 μl water and used entirely for the linear amplification of the polyA-tailed molecules as follows. The eluted DNA was mixed with 2 µl of 10x PCR buffer, 0.4 µl of 10 mM dNTP mix (Invitrogen), 1 µl of 10 µM of a chimeric DNA-RNA oligo-d(T)_50_-r(T)_3_ oligonucleotide (50 2′-deoxythymidine and 3 thymidine nucleotides at the 3′ end) and 1U of Taq DNA polymerase (Tiangen). The amplification conditions were as follows: initial denaturation at 94 °C for 30 s, followed by 10 cycles of denaturation at 94 °C for 1 min, annealing at 55 °C for 30 s and extension at 72 °C for 30 s. After this, the DNA was purified with 2X volumes of VAHTS beads and eluted in 15 μl of water.

polyC tailing: the eluted DNA was denatured at 95 °C for 5 min in 19 µl volume with 2 µl of 10X TdT buffer and 2 µl of 2.5 mM CoCl_2_ followed by rapid snap-cooling on ice. Five units of TdT (NEB) and 2 µl of 10 mM dCTP (Roche) were then added to denatured DNA to the total volume of 22 µl and incubated at 37 °C for 30 min. To block free 3′-OH ends, 2 µl of 10 mM ddCTP (Roche) was added to the tailing mix and incubated at 37 °C for additional 30 min, followed by incubation at 70 °C for 10 min to inactivate TdT.

Library construction: the polyC-tailed DNA was purified with 2X volume of VAHTS beads, eluted in 15.2 μl water and used in its entirety for the library construction as follows. The eluted DNA was subjected to PCR amplification in 20 μl reaction volume containing: 1X PCR buffer, 0.4 µl of 10 mM dNTP mix (Invitrogen), 1 μl of each of the following two oligonucleotides P5G10 (AATGATACGGCGACCACCGAGAtctACACTCTTTCCCTACACGACGCTCTTCCGATCTGGGGGGGGGGHN) and P7T12 (CAAGCAGAAGACGGCATACGAGATcgtgatGTGACTGGAGTTCAGACGTGTGCTCTTCCGATCTTTTTTTTTTTTTVN) each at 10 μM and 1U of Taq DNA polymerase (Tiangen). The PCR conditions were as follows: (1) initial denaturation at 94 °C for 3 min; (2) followed by 1 cycle of denaturation at 94 °C for 30 s, annealing at 55 °C for 1 min and extension at 72 °C for 1 min; (3) 1 cycle of denaturation at 94 °C for 30 s, annealing at 37 °C for 1 min and slow ramp at 2 °C per minute to 72 °C followed by 2 min incubation, followed by (4) 30 cycles at 94 °C for 30 s and extension at 72 °C for 1 min. The amplified DNA was purified with 2X volumes of VAHTS beads and eluted in 20 μl of water. The concentration of DNA was measured using Qubit 3.0 fluorometer and “dsDNA HS Assay” kit (Thermo Fisher Scientific). Sequencing on the Illumina platform was performed using paired-end 150 bp strategy and outsourced to Novogene Corporation (Beijing).

### Processing of raw SMS and Illumina reads and mapping SSBs

SMS: First, raw SMS reads were filtered for length (≥25 bases) and sequence quality using Helisphere package as previously described^51^. Second, the remaining reads were then aligned to the GRCh37/hg19 assembly of the human genome using indexDPgenomic software^52^. To estimate enrichment in repeat classes, we used uniquely and non-uniquely aligning reads. Since a read from the latter category had multiple alignments with the same scores (non-unique reads), one such alignment was randomly chosen for analysis. For all other analyses, only reads with unique alignments in the genome were used. Third, the alignments likely arising from internal priming to A-rich sequences in the genome were removed. Since the strand of an alignment represents strand opposite to the one with the break, polyA-rich regions on the SSB strand would be represented by polyT-rich regions on the strand of the alignments. Thus, alignments where fraction of T’s in 20 base 5′ upstream sequence was >40% were removed from the downstream analysis. Finally, since the Helicos SMS sequencing requires fill-n-lock step where the first base after the polyA tail is used to visualize the templates and thus not sequenced^50^, the 5′ coordinates of each post-filtration alignment were extended by 1 base. The coordinates of an SSB were defined as coordinates of one base corresponding to the 5 prime-most base of an alignment. For example, if coordinates of an aligned read were the chr17: 7,677,036-7,577,064 on “+” strand, then the coordinates of the corresponding SSB were chr17: 7,677,035-7,677,036 on “−” strand of the genome.

Illumina: (1) Only paired-end raw reads where read 1 started with 10G’s, read 2 started with 12T’s and each base of each read had Phred quality score >20 were selected. (2) Such reads were then aligned to the GRCh37/hg19 or GRCm38/mm10 assemblies of human or mouse genomes respectively using BWA-MEM (Version 0.7.12) with default settings. Only pairs of reads where both reads 1 and 2 uniquely mapped to the genome with appropriate configuration and spacing were kept. (3) Similar to the SSiNGLe-SMS data, pairs of reads where fraction of T’s in the 20-base 5′ upstream sequence in the read 2 alignment was >40% were removed from the downstream analyses. An SSB was defined as the first base after 12T’s in the read 2. For the detection of the 7S DNA species (Fig. 2f, g, Supplementary Tables 2, 3) reads were mapped only to the chrM sequence.

SMS and Illumina: (1) Breaks found in the un-tailed samples were removed. (2) Finally, with the exception of analyses of overlap with the repetitive elements and restriction enzyme cut sites, breaks found by SMS or Illumina reads mapping to repeats as defined by the RepeatMasker were removed.

### Bioinformatics analysis

The R environment^53^ also used for all bioinformatics analyses below. Overlaps between SSBs and various genomic elements were tested using the following tracks and tables downloaded from the GRCh37/hg19 assembly of the UCSC genome browser^54^ (http://hgdownload.soe.ucsc.edu/goldenPath/hg19/database/):

1. RepeatMasker (table rmsk.txt)
2. UCSC Genes (table knownGene.txt)
3. Chromatin State Segmentation by HMM from ENCODE/Broad^33^ for K562 and GM12878 cell lines (respective tables wgEncodeBroadHmmK562HMM.bed and wgEncodeBroadHmmGM12878HMM.bed) including promoters, enhancers and insulators
4. Histone Modifications by ChIP-seq from ENCODE/Broad Institute for K562 cell line (http://www.genome.ucsc.edu/cgi-bin/hgFileUi?db=hg19&g=wgEncodeBroadHistone)
5. Replication signal for K562 cell line (http://www.genome.ucsc.edu/cgi-bin/hgFileUi?db=hg19&g=wgEncodeUwRepliSeq)
6. Nucleosome signal for K562 cell line (http://www.genome.ucsc.edu/cgi-bin/hgFileUi?db=hg19&g=wgEncodeSydhNsome)
7. 1000 Genomes Phase 3 Integrated Variant Calls (table ALL.chr*.phase3.genotype.vcf)
8. 100 species conservation scores, including both PhastCons (http://hgdownload.soe.ucsc.edu/goldenPath/hg19/phastCons100way/hg19.100way.phastCons/chr*.phastCons100way.wig.Fix) and PhyloP (http://hgdownload.soe.ucsc.edu/goldenPath/hg19/phyloP100way/hg19.100way.phyloP100way/chr*.phyloP100way.wigFix.gz).
9. The HeLa DSB raw data from BLESS were downloaded from BLESS supporting website (http://breakome.utmb.edu/Home.html).
10. Coordinates of Top2A cleavage cluster regions were downloaded from NCBI GEO (https://www.ncbi.nlm.nih.gov/geo/query/acc.cgi?acc=GSE79593).

All analyses with the exception of hotspot analyses were based on unique coordinates of breaks. All overlaps were done using exact coordinates of SSBs. However, for overlap analyses with the 1000 Genomes data, the coordinates of SSBs were also extended by ±2, 5 and 10 bp. And, for estimating the ratio of conservation score between nearby regions and positions of SSBs, the coordinates of SSBs were also extended by ±5, 10 and 20 bp. The overlaps between SSBs and the different genomic element were calculated using the “intersect” function of the BEDTools^55^ suite (v2). The enrichment odds ratios and *p*-values of overlaps were calculated using binomial test implemented in the R environment. The *p*-values in Supplementary Data 3 were calculated using one-sided hypergeometric test. The *p*-values and 95% confidence intervals in Supplementary Data 5–7, 10, 14–17, 19 were calculated using two-sided binomial test. All tests based on the Wilcoxon rank-sum were also two-sided. Supplementary Table 6 contains *p*-values, 95% confidence intervals, Cohen’s *d* effect sizes, mean values and standard deviations for the odds ratios reported in Fig. 5 for SSiNGLe-SMS data.

For the GO analysis, genes were first assigned to the ENCODE promoters found in K562 cells within 5 kb from annotated 5′ ends of the genes resulting in 16,620 genes out of 28,514 genes in UCSC Genes database. Then, for each sample a list of genes with three breaks in promoters was selected from the background of 16,620 genes. For the exon GO analysis, genes with three breaks in exons were selected from either 16,620 or all 28,514 genes. For each sample, we selected significant GO terms that were shared by both biological replicas (Supplementary Data 8,11,12). The gene set enrichment analysis was performed using the GOstats^56^ package (version 2.46) in R environment (package org.Hs.eg.db). Supplementary Data 8,11,12 contain GO terms found to have adjusted *p*-values < 1E–4 (hypergeometric test) and odds ratios of >2 in biological replica B1 and *p*-values < 0.01 in the replica B2 of SSiNGLe-ILM analyses of drug-treated K562 cells. If for some samples no GO terms with such criteria could be found, we extracted terms having adjusted *p*-values < 0.01 in both bio-replicas and then selected top three terms based on B1 odds ratios. For the GO term analysis of the SSiNGLe-SMS data, all samples were combined and genes with promoters or exons containing at least 2 reads were selected. GO terms with adjusted *p*-values < 0.01 (hypergeometric test) are shown in Supplementary Data 9 & 13. The logistic and multiple linear regression models were built respectively using “glm” and “lm” functions in R with default parameters. The ROC curve and AUC values were generated using the “ROCR” package. McFadden’s pseudo R^2^ was calculated by the “pscl” package.

### Reporting summary

Further information on research design is available in the Nature Research Reporting Summary linked to this article.

## Supplementary information

Supplementary Information

Supplementary Data 1

Supplementary Data 2

Supplementary Data 3

Supplementary Data 4

Supplementary Data 5

Supplementary Data 6

Supplementary Data 7

Supplementary Data 8

Supplementary Data 9

Supplementary Data 10

Supplementary Data 11

Supplementary Data 12

Supplementary Data 13

Supplementary Data 14

Supplementary Data 15

Supplementary Data 16

Supplementary Data 17

Supplementary Data 18

Supplementary Data 19

Reporting Summary

## Data Availability

Processed data used to make conclusions in the text are presented in Supplementary Tables and Data files and referred to in the appropriate places in the main text, figure legends and Methods. The coordinates of breaks and filtered sequencing data have been deposited to GEO with accession number GSE139011. All data are available from the authors upon reasonable request.

## Code availability

Custom scripts constituting the pipeline used to generate coordinates of breaks from the sequencing reads are available from GitHub at https://github.com/huifencao/DB-Nature_Communications-2019. Downstream bioinformatic analyses based on the coordinates of the breaks were performed using standard packages in the R environment and BEDTools as indicated in Methods.
