# Supplementary material for: Novel approach reveals genomic landscapes of single-strand DNA breaks with nucleotide resolution in human cells
Source: Nat Commun. 2019 Dec 20;10:5799. doi: 10.1038/s41467-019-13602-7 (PMC6925131; doi:10.1038/s41467-019-13602-7)
Supplement: Supplementary file 1 — Supplementary Information [file 41467_2019_13602_MOESM1_ESM.pdf]

# Supplementary Information

# Novel approach reveals genomic landscapes of single-strand DNA breaks with nucleotide resolution in human cells

**Cao et al.**

## Supplementary Note 1

*Detection of endogenous breaks induced by expression of site-specific endonuclease*

We generated stable K562 cell line expressing doxycycline (Dox) inducible version of nuclear-targeted AsiSI endonuclease. We performed induction of this enzyme under different conditions: in addition to standard Dox induction, we used Dox in combination with (1) inhibitor of CpG methylation decitabine since AsiSI is blocked by this type of modification, (2) cocktail of inhibitors of DNA repair kinases ATM, ATR and DNA-PK to inhibit repair of the AsiSI-induced breaks and thus potentially improve their detection, and (3) mixture of decitabine and the kinase inhibitors (Materials and Methods). For each treatment, we had corresponding -Dox control. We then compared break profiles in and around the expected cut sites in +Dox vs -Dox controls. Overall, we tried 14 different pairs of +Dox and -Dox samples and in none of them we could detect significant signal at the cut sites or in their immediate vicinity in the AsiSI-induced samples. These results suggested that *in vivo*, un-repaired breaks induced by this enzyme are quite rare, consistent with inefficient cleavage of this enzyme *in vivo*[1]. As the next step, we combined all 28 samples to generate two groups of +Dox and -Dox samples containing respectively 34,323,171 and 37,859,166 breaks total of which correspondingly 411 and 355 mapped to +/- 10 bp from the expected cut sites. This translated into 1.27-fold enrichment in the +Dox group after normalization for total number of breaks with p-value 0.008 (Wilcoxon signed-rank test) (Figure 2H, Supplementary Data 4).

*Overlap with various histone modifications and chromatin modulating factor binding sites*

We next analyzed overlap between SSBs and genomic regions bound by 11 different histone modifications and 6 chromatin modulating factors profiled in K562 by the ENCODE consortium[2] (Supplementary Data 7). We observed the most significant overlap with histone marks associated with active chromatin regions: H3K4me2 (odds ratios 1.02-3.26, median 1.98, p-value < 8.0E-02), H3K27Ac (odds ratios 1.01-2.85, median 1.79, p-value <2.1-15), H3K4me3 (odds ratios 0.91-2.85, median 1.80, p-value <2.2E-16) and H3K9Ac (odds ratios 0.93-2.45, median 1.64, p-value <2.9E-16) (Supplementary Data 7). We observed the least significant overlap with the repressive chromatin mark H3K9me3 (odds ratios 0.52-0.67 median 0.6 p-value <2.2E-16) (Supplementary Data 7). Consistent with the enrichment in insulators, we observed the highest overlap with the insulator protein CTCF of all chromatin modulating factors, (odds ratios 1.11-2.91, median 1.9, p-value <2.2E-16) (Supplementary Data 7).

*Overlap with DSBs*

As mentioned above, DSBs detected by our method had a statistically-significant overlap with DSBs found in the BLESS protocol. Furthermore, the SSB profiles from the 3 different biological replicas of HeLa cells also had statistically-significant overlap with the DSBs found by the BLESS protocol. The overlap was extremely significant and reproducible when the precise coordinates of SSBs and DSBs were overlapped within the satellite repeats (odds ratios of 74-93 for the 3 replicas,

54 for merged data) and it gradually decreased with increasing distance between the two types of breaks (Figure 2D) (Supplementary Data 3). However, we could also observe statistically-significant overlap outside of the satellite repeat regions, however, with much lower odds ratios of 1.12-1.26 depending on the distance between the SSBs and DSBs (Figure 2D, Supplementary Data 3). Overall, the number of SSBs overlapping BLESS breaks significantly outnumbered the DSBs found by SSiNGLe-ILM and overlapping the latter (Figure 2D). For example, 448 DSBs and 1225 SSBs overlapped BLESS breaks with exact matches in the satellite repeats and correspondingly 27 and 1048 in non-satellite regions. However, as expected, the odds ratios of overlap between the SSiNGLe-ILM DSBs were consistently higher those of SSBs (Figure 2D), since BLESS is designed to detect the former.

*Evolutionary sequence conservation of breaks*

If DNA breaks cause greater sequence variability, then the nucleotide positions corresponding to them would be expected to have less evolutionary conservation than neighboring sequences. We tested this by calculating ratios of the PhastCons scores for nucleotide positions corresponding to each break vs average conservation score of the flanking sequences defined as +/-5, 10 and 20 bp windows around the break. Then, we calculated average ratios for every sample and found them to always be below 1 for every window size (Supplementary Table 5). Average ratios across all K562 samples were 0.69, 0.72 and 0.77 for +/-5, 10 and 20 bp windows respectively for SSiNGLe-ILM. The corresponding values for SSiNGLe-ILM with no formaldehyde were 0.68, 0.72 and 0.77 and SSiNGLe-SMS - 0.70, 0.76 and 0.82. Consistent with the variant overlap data above, these results strongly indicate that genomic positions corresponding to DNA breaks do have less evolutionary conservation than neighboring sequences.

*Deep sequencing and hotspots of breaks*

The results described above are based on 0.18-1.75M (median 0.57) filtered reads per sample. We then explored the landscape of breaks by sequencing 6 samples (romidepsin and DMSO treatments, 6, 24 and 48hr) at higher depths of 15M filtered reads each. The ratio of unique breaks to total number of filtered reads indicative of "breakome" complexity decreased with the increase in sequencing depth as expected (Figure 3H). Interestingly, the higher complexities were found at romidepsin 24 and 48h vs 6h and DMSO at 48h vs 6 and 24h (Figure 3H), potentially coinciding with the onset of apoptotic DNA fragmentation in the later timepoint samples. While as shown above, the method can detect DSBs, majority of the observed signal (98-99%) did not overlap on both strands within +/- 2 bp using the deep sequencing data, suggesting that as expected, the vast majority of the signal was contributed by SSBs.

While on average 79% of the deep-seq breaks were represented by single reads, 0.9-4.2% were found by ≥4 reads, thus potentially representing hotspots of SSBs. Such hotspots had a number of interesting features, many of them could be defined by stronger magnitude of the patterns described above. First, the hotspots had higher enrichment in the satellite repeats compared to the

singletons (average odds ratio of 9.9 vs 1.8, p-value <2.2E-16). Second, they had higher enrichments in exons and promoters (Supplementary Figure 3 A &B). Third, the difference in the template/non-template ratios for exons and introns was more significant ((Supplementary Figure 3C). Fourth, the increase in the odds ratio of overlap between the hotspots and genomic elements (promoters, exons, enhancers and insulators in particular) at the later stages of drug treatment was higher than that of singleton breaks (Supplementary Figure 3D). Finally, the overlap with genomic variants was also higher for the most part (Supplementary Figure 3E).

*Effect of formaldehyde crosslinking*

Importantly, as shown above, we observed the same trends of the enrichments in the regulatory regions, exons and introns as well as of the template/non-template ratios, association with sequence variants and replication timing distribution in the samples without the formaldehyde crosslinking step (Figure 3, Supplementary Data 1, 5-7, 10, 14-17, 19) arguing that the observed results are not artifacts of the crosslinking.

*Genomic patterns are shared by multiple cell types*

Considering the wide popularity of the liquid biopsy diagnostics e.g. venous blood sampling, we initially tested whether our method can detect SSBs in human PBMCs isolated from 84 female donors aged 20-89 years. Of those, 18 were profiled using SSiNGLe-SMS and 44 with SSiNGLe-ILM and 22 with both techniques. As shown in Figure 2B, both SSiNGLe-SMS and SSiNGLe-ILM showed significantly higher signal compared to the un-tailed samples arguing that DNA breaks can be reliably detected in this cell type. Then, we investigated whether genomic patterns of SSBs from these samples had enrichment in various genomic features found in K562. Strikingly, SSBs from both sample types shared a number of key genomic patterns, including enrichment in functional elements and association with sequence polymorphisms. As in K562, the same patterns were detected in PBMCs using both SSiNGLe-SMS and SSiNGLe-ILM.

***First***, the PBMC breaks were enriched in exons and introns with corresponding odds ratios of 1.29-2.14 (p-value <2.2E-16) and 1.25-1.3 (p-value <2.2E-16) using SSiNGLe-SMS and 1.31-2.52 (p-value <2.2E-16) and 1.14-1.19 (p-value <2.2E-16) using SSiNGLe-ILM (Figure 3B, Supplementary Data 10). ***Second***, the ratios of PBMC breaks mapping to template vs non-template strand were also consistently higher for exons than introns, with the corresponding ranges of 0.98-1.08 and 0.88-0.95 using SSiNGLe-SMS and 1.00-1.44 and 0.89-0.98 using SSiNGLe-ILM (Figure 3D, Supplementary Data 14). These differences between exons and introns were statistically significant across all PBMCs samples (p-value 1.43E-14 SSiNGLe-SMS and <2.2E-16 SSiNGLe-ILM, Wilcoxon rank-sum test). Moreover, the differences in the template/non-template ratios could also be found for HeLa and N2A cell lines (Figure 3D). ***Third***, PBMC SSBs were enriched with odds ratios of 1.02-1.5, 0.92-1.75 and 0.97-1.20 in respectively insulators, promoters and enhancers using SSiNGLe-SMS and 1.12-1.70, 0.99-1.89 and 1.00-1.25 using SSiNGLe-ILM (Figure 3B, Supplementary Data 6). For this analysis, we used regulatory regions

from an immortalized B-cell GM12878 derived using the same computation approach as the ones from K562 to test for enrichment of SSBs from PBMCs. The lower odds ratios in PBMCs compared to K562 likely reflect the fact that the regulatory elements were not derived from the former, but rather from one cell-type of this complex tissue, and also inter-individual variation as discussed below. ***Fourth***, the PBMC breaks were also significantly associated with SNPs using SSiNGLe-SMS and SNPs and indels using SSiNGLe-ILM (Figure 3F, Supplementary Data 16-17) and also showed lower sequence conservation than the neighboring sequences with very similar conservation ratios of breaks vs flanking sequences as in K562 (Supplementary Table 5). ***Finally***, satellite repeats represented the class of repeats most significantly associated with the PBMC breaks: odds ratio 5.86 with SSiNGLe-SMS and median odds ratio 6.17 with SSiNGLe-ILM, p-value <2.2E-16 for each sample (Supplementary Data 5).

*Breaks tend to occur in promoters and exons of genes with specific functions*

The ability of the "breakome" to represent the molecular state of the cell - particularly the pattern of enrichment of breaks in promoters and exons - prompted us to investigate whether this enrichment was random across all genes or specific to genes with certain biochemical functions. To answer this, we performed Gene Ontology (GO) analysis on genes with breaks in either promoters or exons. For the former analysis, genes were first assigned to the ENCODE promoters found in K562 cells within 5kb from annotated 5' ends of the genes resulting in 16,620 genes out of 28,514 genes in UCSC Genes database. Then, for each sample a list of genes with 3 breaks in promoters was selected from the background of 16,620 genes. For the latter analysis, genes with 3 breaks in exons were selected from either 16,620 or all 28,514 genes. For each sample, we selected significant GO terms that were shared by both biological replicas (Supplementary Data 8, 11-12).

Both analyses revealed consistent patterns of enrichment of specific biological functions (Supplementary Data 8, 11-12). GO terms of genes with breaks in promoters revealed clear and consistent enrichment of terms associated with RNA processing, cell cycle control such as "cell cycle G2/M phase transition" or "cell cycle G1/S phase transition", and DNA repair such as "DNA damage checkpoint" in multiple drugs and in multiple time points (Supplementary Data 8). Surprisingly, the genes with breaks in exons were significantly enriched in neuronal functions, such as "axonogenesis" and "neurogenesis", GTPase signaling, such as "regulation of small GTPase mediated signal transduction" and cytoskeleton, such as "microtubule-based movement". This enrichment was most evident compared to the background of all genes (Supplementary Data 11), however it was also prominent relative to the background of 16,620 genes also used for the promoter GO analysis (Supplementary Data 12). Thus, the enriched GO terms for the genes with breaks in promoters and exons are not explained by different transcriptome backgrounds.

Some of the same terms were also detected on the combined data using SSiNGLe-SMS (Supplementary Data 9,13). For example, the genes with breaks in promoters were enriched in "mitotic cell cycle checkpoint" (p-value 8.8E-06), "RNA processing" (p-value 4E-04) and "DNA

damage checkpoint" (p-value 1.9E-03) GO terms (Supplementary Data 9). Likewise, genes with breaks in exons were enriched in the "neurogenesis" term (p-value 6.2E-06) (Supplementary Data 13). Interestingly, temporal profiles revealed further differences between genes with breaks in promoters and exons (Supplementary Figure 4). For example, the significance of enrichment of GO terms among genes with breaks in promoters increased significantly at 24-48h time points, often peaking at 36h (Supplementary Figure 4A). This paralleled strong increase of odds ratios of co-occurrence of breaks in promoters in these timepoints (Figure 3C), suggesting that the observed increase in breaks favored promoters of genes with specific functions. On the other hand, enrichment of functions in genes with breaks in exons was relatively constant across multiple time points of treatments (Supplementary Figure 4B).

Temporal profiles also revealed difference among drugs. First, despite multiple shared GO terms among different drug treatments, their temporal patterns were often specific to particular drugs (Supplementary Figure 4, Supplementary Data 8, 11-12). For example, at 48h timepoint, the "DNA damage checkpoint" term was enriched in imatinib, but not in other drugs (Supplementary Figure 4A). Likewise, "microtubule based movement" was enriched only at 12 and 24h timepoints in SN-38, while it was enriched at most time points for all other treatments (Supplementary Figure 4B). All in all, these analyses demonstrate that the global pattern of SSBs – the "breakome" – is not random, but rather represents a specific and reproducible fingerprint reflective of the biological state of the cell.

## Supplementary References


1. Iacovoni JS, Caron P, Lassadi I, Nicolas E, Massip L, Trouche D, *et al.* High-resolution profiling of gammaH2AX around DNA double strand breaks in the mammalian genome. *The EMBO journal* 2010, **29**(8)**:** 1446-1457.

2. Consortium EP, Dunham I, Kundaje A, Aldred S, Collins P, Davis C, *et al.* An integrated encyclopedia of DNA elements in the human genome. *Nature* 2012, **489:** 57 - 74.

## Supplementary Figures

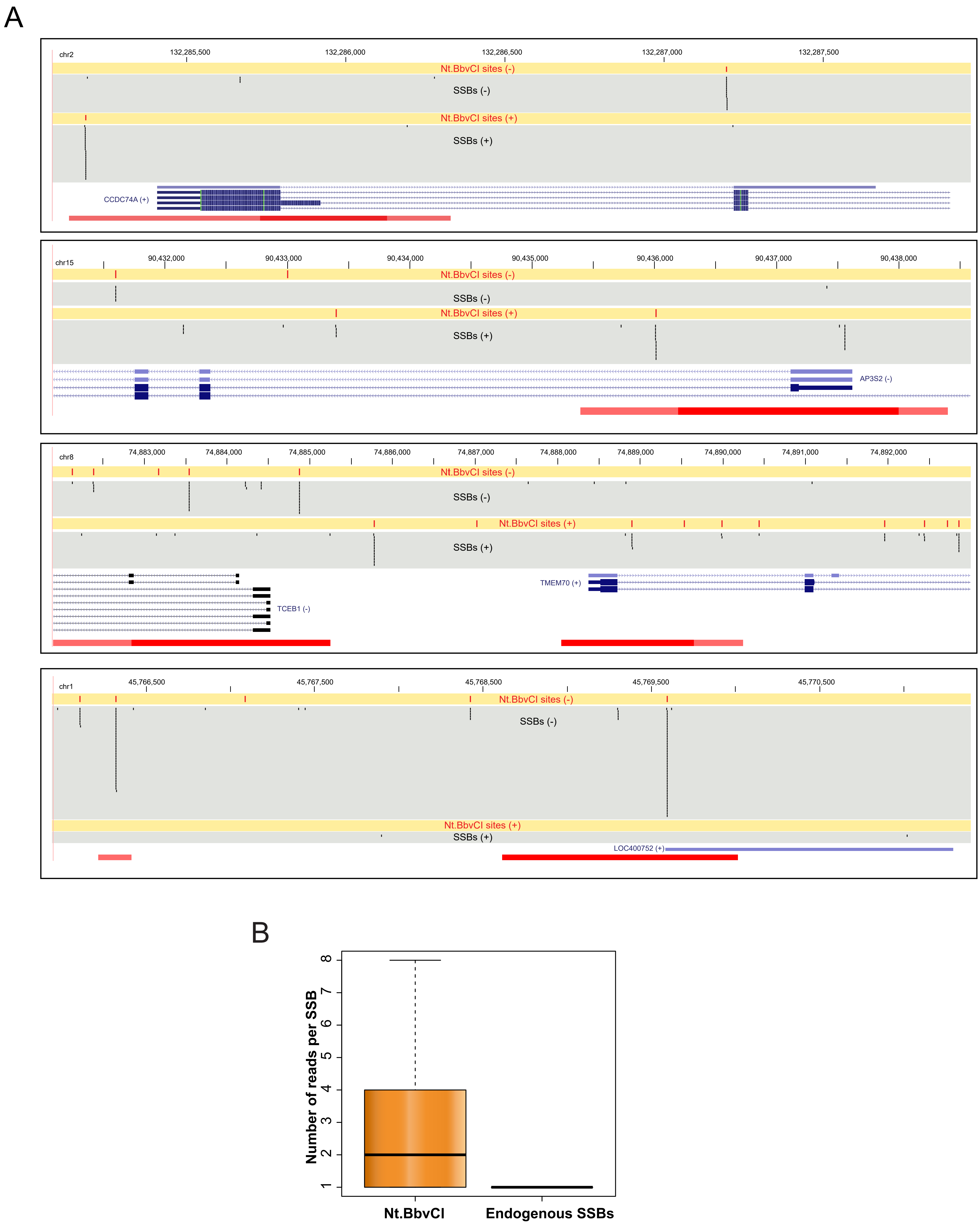


**Supplementary Figure 1. Detection of cleavage at Nt.BbvCI sites in deep sequencing data.** (a) Positions of Nt.BbvCI sites and SSBs are shown for each strand of the genome for 4 different genomic regions. Red bars below the UCSC Genes represent K562 promoter regions as defined by Chromatin State Segmentation by HMM from ENCODE/Broad. Breaks not overlapping with the Nt.BbvCI sites represent endogenous SSBs present in these samples. (b) Box plots of depth of detection (reads per SSB) for the Nt.BbvCI sites and endogenous SSBs.

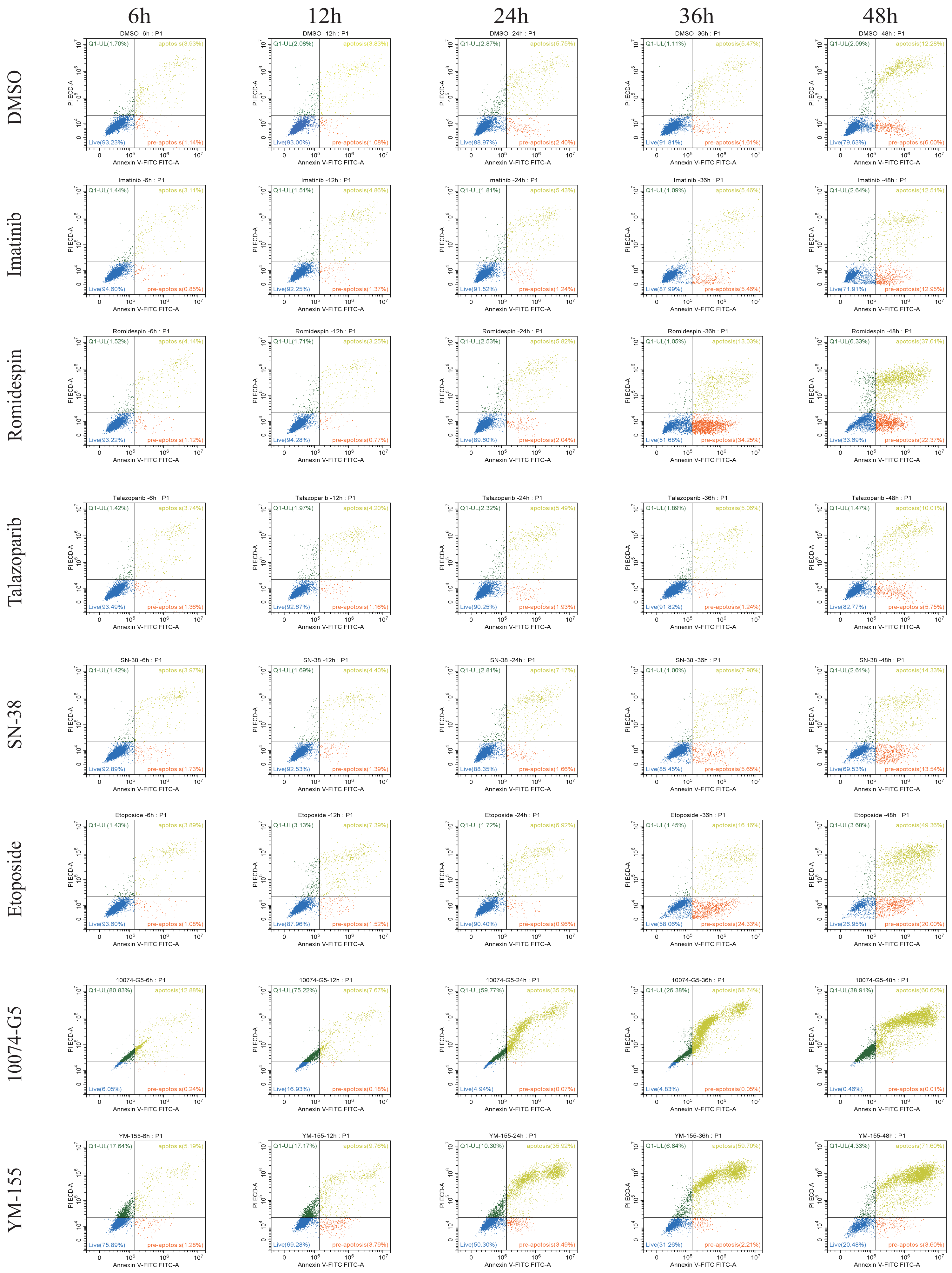


**Supplementary Figure 2. Flow sorter detection of apoptotic K562 cells under various treatments.** Apoptotic cells were detected based on fluorescent signal intensity from FITC-conjugated AnnexinV (FITC channel) (X-axes) using gates represented by thin vertical lines. Y-axes represent fluorescent signal intensity from PI staining (ECD channel).

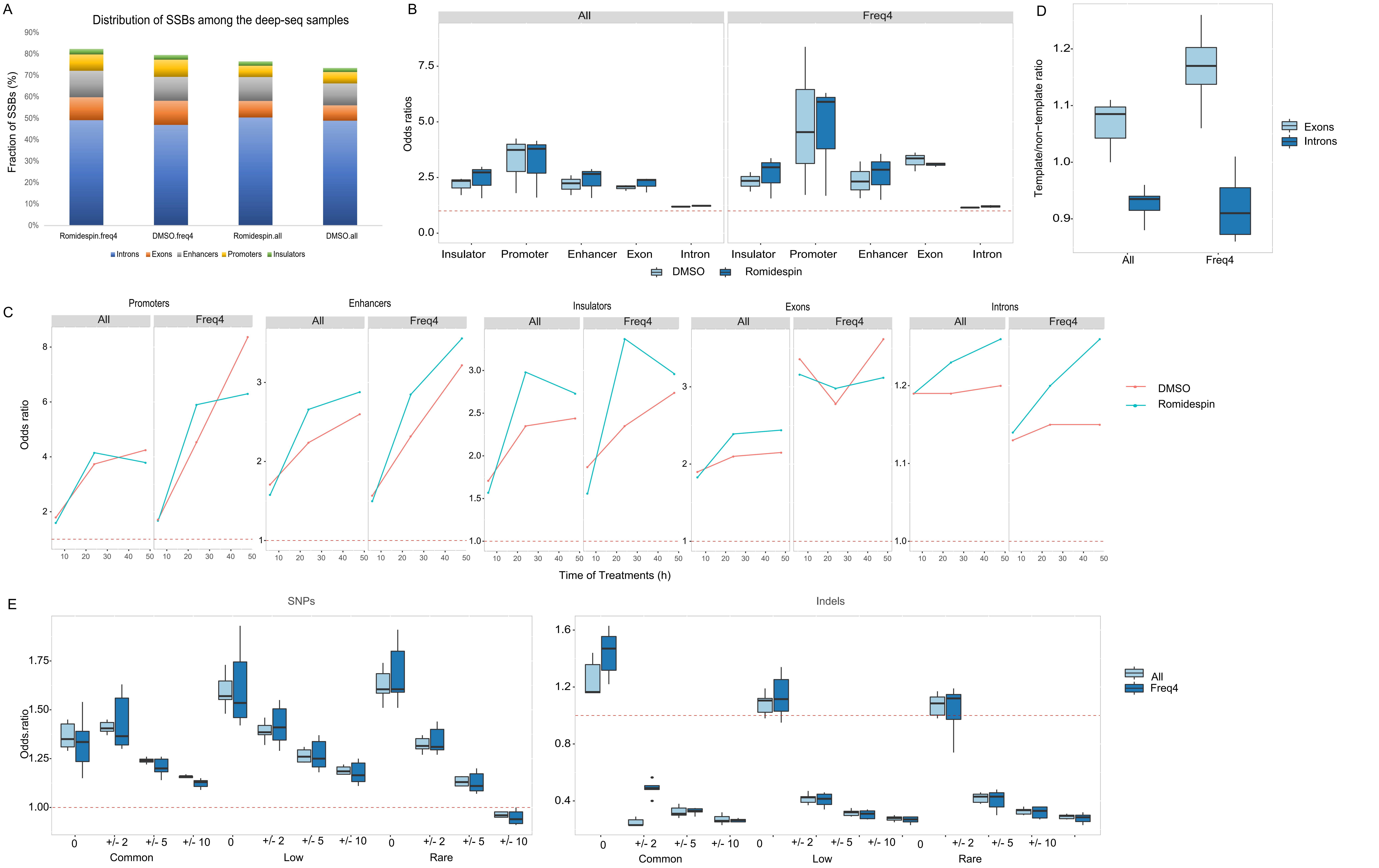


**Supplementary Figure 3. Genomic landscape of SSB hotspots.** Results for all breaks and hotspots detected by ≥4 reads ("Freq4") based on deep sequencing of romidepsin and DMSO (6, 24 and 48hr) samples are shown as follows.
(a) Average fraction of SSBs falling within each of the indicated genomic elements.
(b) Box plots of distributions of odds ratios of overlap among different genomic elements.
(c) Distribution of odds ratios of overlap among different genomic elements (Y-axes) and drug treatment times (X-axes) (d) Box plots of distributions of template/non-template ratios for exons and introns.
(e) Box plots of distribution of odds ratios of overlap among SNPs and indels (common, low and rare frequency).

A

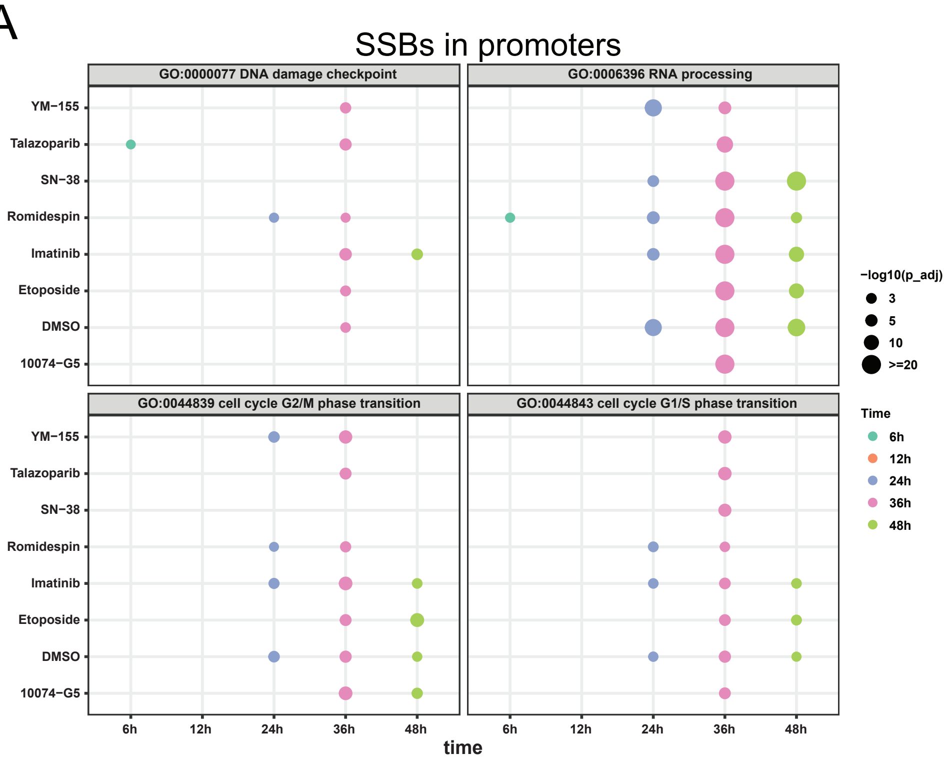


B

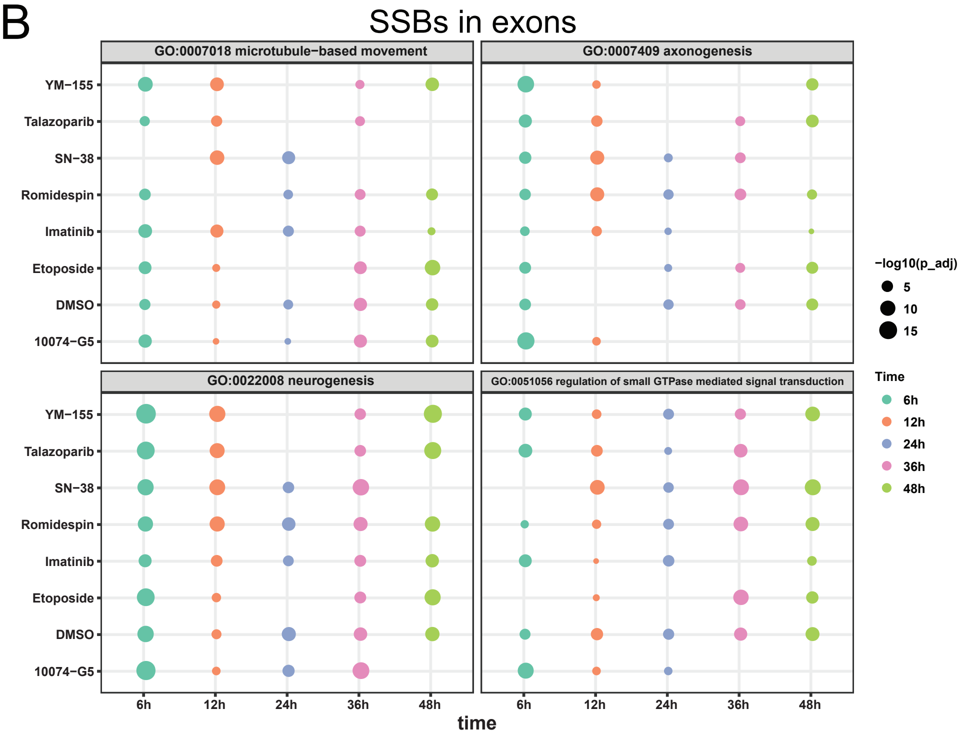


**Supplementary Figure 4. Temporal profiles of enrichment of specific GO terms in genes with breaks in promoters and exons.** For each indicated GO term, adjusted p-values are shown in -log10 space for genes with breaks in (a) promoters and (b) exons for each drug and time point. Samples with the adjusted p-values of <0.01 in replica B1 are shown. The corresponding p-values for replica B2 are shown in Supplementary Data 8, 11-12.

# Supplementary Tables

**Supplementary Table 1. Summary of reads mapped to human genome in human-mouse mixture experiments**

| Dilution (K562 : N2A) | No. of clean Reads | No. of unique reads mapped to HG19 | Ratio of Mapping | Uniquely mapping reads filtered for polyA-stretches | % Post-Filtered |
|---|---|---|---|---|---|
| K:N 1:2 | 1347501 | 457956 | 34% | 290552 | 63.45 |
| K:N 1:4 | 1134690 | 214385 | 19% | 132844 | 61.97 |
| K:N 1:9 | 1102285 | 179195 | 16% | 112358 | 62.70 |

**Supplementary Table 2. The percentage of total reads mapped to mitchondrial genome (median for each sample type)**

| Cell Type | Number of reads mapped to chrM | Number of total reads | % Reads mapped to chrM |
|---|---|---|---|
| PBMC | 1598 | 2797119 | 0.06 |
| HeLa | 27606 | 2294418 | 1.20 |
| K562.B1 | 9957 | 2239693 | 0.44 |
| K562.B2 | 5708 | 1560712 | 0.37 |

**Supplementary Table 3. The fraction of reads mapping to mitochondrial genome mapping in the vicinity of the 3' end of the 7S DNA molecule**

| chrM coordinate | PBMC | | HeLa | | K562.B1 | | K562.B2 | |
|---|---|---|---|---|---|---|---|---|
| | % Reads (- strand) | % Reads (+ strand) | % Reads (- strand) | % Reads (+ strand) | % Reads ( - strand) | % Reads ( + strand) | % Reads ( - strand) | % Reads ( + strand) |
| 16096 | 0.00 | 0.00 | 0.05 | 0.00 | 0.03 | 0.00 | 0.00 | 0.00 |
| 16097 | 0.00 | 0.00 | 0.04 | 0.00 | 0.02 | 0.00 | 0.06 | 0.01 |
| 16098 | 0.00 | 0.00 | 0.00 | 0.00 | 0.00 | 0.00 | 0.00 | 0.00 |
| 16099 | 0.00 | 0.00 | 0.02 | 0.00 | 0.00 | 0.00 | 0.00 | 0.00 |
| 16100 | 0.00 | 0.00 | 0.00 | 0.00 | 0.00 | 0.00 | 0.00 | 0.00 |
| 16101 | 0.00 | 0.00 | 0.00 | 0.00 | 0.00 | 0.00 | 0.00 | 0.00 |
| 16102 | 0.00 | 0.00 | 0.00 | 0.00 | 0.00 | 0.00 | 0.00 | 0.00 |
| 16103 | 0.00 | 0.00 | 0.00 | 0.00 | 0.00 | 0.00 | 0.00 | 0.00 |
| 16104 | 0.00 | 0.00 | 0.09 | 0.00 | 0.02 | 0.00 | 0.00 | 0.00 |
| 16105 | 0.55 | 0.00 | 0.83 | 0.00 | 0.26 | 0.00 | 0.42 | 0.00 |
| 16106 | 2.59 | 0.00 | 3.37 | 0.00 | 1.03 | 0.00 | 0.74 | 0.00 |
| 16107 | 40.82 | 0.00 | 45.96 | 0.00 | 16.87 | 0.00 | 12.90 | 0.00 |
| 16108 | 13.84 | 0.00 | 16.43 | 0.00 | 7.98 | 0.01 | 6.17 | 0.00 |
| 16109 | 0.45 | 0.00 | 12.37 | 0.00 | 0.48 | 0.01 | 0.41 | 0.00 |
| 16110 | 0.32 | 0.00 | 2.13 | 0.00 | 0.40 | 0.00 | 0.28 | 0.00 |
| 16111 | 0.41 | 0.00 | 1.28 | 0.00 | 0.38 | 0.00 | 0.34 | 0.00 |
| 16112 | 0.51 | 0.00 | 0.85 | 0.00 | 0.70 | 0.00 | 0.66 | 0.00 |
| 16113 | 0.08 | 0.00 | 0.19 | 0.00 | 0.08 | 0.00 | 0.09 | 0.00 |
| 16114 | 0.04 | 0.00 | 0.08 | 0.00 | 0.05 | 0.00 | 0.02 | 0.00 |
| 16115 | 0.10 | 0.00 | 0.15 | 0.00 | 0.09 | 0.00 | 0.08 | 0.00 |
| 16116 | 0.02 | 0.00 | 0.03 | 0.00 | 0.02 | 0.00 | 0.01 | 0.00 |

**Supplementary Table 4. Proportion of apoptotic cells in each sample**

| Sample ID | % apoptotic cells |
|---|---|
| Imatinib-6h | 4.0% |
| Imatinib-12h | 6.2% |
| Imatinib-24h | 6.7% |
| Imatinib-36h | 10.9% |
| Imatinib-48h | 25.5% |
| 10074-G5-6h | 13.1% |
| 10074-G5-12h | 7.9% |
| 10074-G5-24h | 35.3% |
| 10074-G5-36h | 68.8% |
| 10074-G5-48h | 60.6% |
| YM-155-6h | 6.5% |
| YM-155-12h | 13.6% |
| YM-155-24h | 39.4% |
| YM-155-36h | 61.9% |
| YM-155-48h | 75.2% |
| Talazoparib-6h | 5.1% |
| Talazoparib-12h | 5.4% |
| Talazoparib-24h | 7.4% |
| Talazoparib-36h | 6.3% |
| Talazoparib-48h | 15.8% |
| SN-38-6h | 5.7% |
| SN-38-12h | 5.8% |
| SN-38-24h | 8.8% |
| SN-38-36h | 13.6% |
| SN-38-48h | 27.9% |
| Etoposide-6h | 5.0% |
| Etoposide-12h | 8.9% |
| Etoposide-24h | 7.9% |
| Etoposide-36h | 40.5% |
| Etoposide-48h | 69.4% |
| Romidespin-6h | 5.3% |
| Romidespin-12h | 4.0% |
| Romidespin-24h | 7.9% |
| Romidespin-36h | 47.3% |
| Romidespin-48h | 60.0% |
| DMSO-6h | 5.1% |
| DMSO-12h | 4.9% |
| DMSO-24h | 8.2% |
| DMSO-36h | 7.1% |
| DMSO-48h | 18.3% |

**Supplementary Table 5. The conservation ratios (SSB Position vs Window including indicated flanking sequence) of merged K562 and PBMC samples based on PhastCons**

| | SSiNGLe-SMS | | SSiNGLe-ILM | | |
|---|---|---|---|---|---|
| | K562 | PBMC | K562 | K562 with non.formaldehyde | PBMC |
| +/- 5 bp | 0.70 | 0.71 | 0.69 | 0.68 | 0.69 |
| +/- 10 bp | 0.76 | 0.76 | 0.72 | 0.72 | 0.74 |
| +/- 20 bp | 0.82 | 0.82 | 0.77 | 0.77 | 0.79 |

Note: SSiNGLe-SMS results are based on all merged data and SSiNGLe-ILM results are based on the average of individual sample

**Supplementary Table 6. The statistics for Figure 5**

| Wilcoxon rank-sum Test (single sided) in Figure 5 for the SSiNGLe-SMS data | Type | P-value | Mean ( Ages ≥ 60) | Mean ( Ages < 60) | Sd_pooled | Effect Size (Cohen's d) |
|---|---|---|---|---|---|---|
| 1 | Odds Ratio for Exons: Ages < 60 vs Ages ≥ 60 | 2.45E-04 | 1.73 | 1.46 | 0.25 | 1.07 |
| 2 | Odds Ratio for Introns: Ages < 60 vs Ages ≥ 60 | 7.47E-04 | 1.28 | 1.26 | 0.01 | 0.99 |
| 3 | Odds Ratio for Insulators: Ages < 60 vs Ages ≥ 60 | 5.41E-04 | 1.29 | 1.17 | 0.12 | 1.00 |
| 4 | Odds Ratio for Promoters: Ages < 60 vs Ages ≥ 60 | 8.01E-05 | 1.38 | 1.09 | 0.25 | 1.15 |
| 5 | Odds Ratio for Enhancers: Ages < 60 vs Ages ≥ 60 | 1.94E-02 | 1.09 | 1.02 | 0.07 | 1.03 |
| 6 | Phylop.pos with window 40bp: Ages < 60 vs Ages ≥ 60 | 1.52E-03 | 0.96 | 0.94 | 0.03 | 0.63 |
| 7 | Phylop.neg with window 40bp: Ages < 60 vs Ages ≥ 60 | 2.17E-04 | (0.69) | (0.67) | 0.02 | (0.96) |
| 8 | Template ratio of exons vs template ratio of introns: Ages < 60 vs Ages ≥ 60 | 3.88E-04 | 1.12 | 1.16 | 0.03 | (1.01) |
