# Supplementary material for: Novel approach reveals genomic landscapes of single-strand DNA breaks with nucleotide resolution in human cells
Source: Nat Commun. 2019 Dec 20;10:5799. doi: 10.1038/s41467-019-13602-7 (PMC6925131; doi:10.1038/s41467-019-13602-7)
Supplement: Supplementary file 21 — Reporting Summary [file 41467_2019_13602_MOESM21_ESM.pdf]

nature research

Corresponding author(s): Philipp Kapranov, Claes Wahlestedt

Last updated by author(s): Oct 19, 2019

Reporting Summary

Nature Research wishes to improve the reproducibility of the work that we publish. This form provides structure for consistency and transparency in reporting. For further information on Nature Research policies, see Authors & Referees and the Editorial Policy Checklist.

Statistics

For all statistical analyses, confirm that the following items are present in the figure legend, table legend, main text, or Methods section.

n/a	Confirmed	
☐	☒	The exact sample size (*n*) for each experimental group/condition, given as a discrete number and unit of measurement
☐	☒	A statement on whether measurements were taken from distinct samples or whether the same sample was measured repeatedly
☐	☒	The statistical test(s) used AND whether they are one- or two-sided<br>*Only common tests should be described solely by name; describe more complex techniques in the Methods section.*
☒	☐	A description of all covariates tested
☐	☒	A description of any assumptions or corrections, such as tests of normality and adjustment for multiple comparisons
☐	☒	A full description of the statistical parameters including central tendency (e.g. means) or other basic estimates (e.g. regression coefficient) AND variation (e.g. standard deviation) or associated estimates of uncertainty (e.g. confidence intervals)
☐	☒	For null hypothesis testing, the test statistic (e.g. F, t, r) with confidence intervals, effect sizes, degrees of freedom and P value noted<br>*Give P values as exact values whenever suitable.*
☒	☐	For Bayesian analysis, information on the choice of priors and Markov chain Monte Carlo settings
☒	☐	For hierarchical and complex designs, identification of the appropriate level for tests and full reporting of outcomes
☐	☒	Estimates of effect sizes (e.g. Cohen's d, Pearson's r), indicating how they were calculated

Our web collection on statistics for biologists contains articles on many of the points above.

Software and code

Policy information about availability of computer code

Data collection	Helisphere package was used to process SMS reads and align them to the genome. Illumina reads were aligned with BWA-MEM.
Data analysis	R environment (v 3.4) and BEDTools suite (v2) were used for data analysis.

For manuscripts utilizing custom algorithms or software that are central to the research but not yet described in published literature, software must be made available to editors/reviewers. We strongly encourage code deposition in a community repository (e.g. GitHub). See the Nature Research guidelines for submitting code & software for further information.

Data

Policy information about availability of data

All manuscripts must include a data availability statement. This statement should provide the following information, where applicable:

- Accession codes, unique identifiers, or web links for publicly available datasets
- A list of figures that have associated raw data
- A description of any restrictions on data availability

Processed data used to make conclusions in the text are presented in Supplemental Tables and Data files and referred to in the appropriate places in the main text, figure legends and Methods. The coordinates of breaks and filtered sequencing data have been deposited to GEO with accession number GSE139011.

Field-specific reporting

Please select the one below that is the best fit for your research. If you are not sure, read the appropriate sections before making your selection.

☒ Life sciences ☐ Behavioural & social sciences ☐ Ecological, evolutionary & environmental sciences

For a reference copy of the document with all sections, see nature.com/documents/nr-reporting-summary-flat.pdf

Life sciences study design

All studies must disclose on these points even when the disclosure is negative.

Sample size	No sample-size calculation was performed. The sufficiency of sample-size was based on achieving statistical significance of conclusions reported in this study.
Data exclusions	Any sequencing sample that did not pass standard sequencing QC metrics was excluded
Replication	SSB-SMS was performed on one independent biological replicas of K562 drug treatments, SSB-ILM was performed on two additional independent replicas and SSB-ILM with no formaldehyde was performed on another independent replica. HeLa and N2A results are based on 3 independent biological replica each. Nt.BbvCI and AsiSI digests analyses were performed on respectively 3 and 2 independent replicas. 22 out of 40 PBMC samples were profiled using both SSB-SMS and SSB-ILM.
Randomization	Not applicable to this work.
Blinding	Not applicable to this work.

Reporting for specific materials, systems and methods

We require information from authors about some types of materials, experimental systems and methods used in many studies. Here, indicate whether each material, system or method listed is relevant to your study. If you are not sure if a list item applies to your research, read the appropriate section before selecting a response.

Materials & experimental systems

n/a	Involved in the study	
☒	☐	Antibodies
☐	☒	Eukaryotic cell lines
☒	☐	Palaeontology
☒	☐	Animals and other organisms
☐	☒	Human research participants
☒	☐	Clinical data

Methods

n/a	Involved in the study	
☒	☐	ChIP-seq
☐	☒	Flow cytometry
☒	☐	MRI-based neuroimaging

Eukaryotic cell lines

Policy information about cell lines

Cell line source(s)	Human leukemia K562 cell line was obtained from Cell Bank of Chinese Academy of Sciences; mouse neuroblastoma (N2a) and human cervical carcinoma HeLa cell lines were obtained from National Infrastructure of Cell Line Resource.
Authentication	All cell lines were authenticated using microscopical visualization and K562 was further authenticated using RNAseq analysis
Mycoplasma contamination	The cell lines were not tested for mycoplasma contamination
Commonly misidentified lines (See ICLAC register)	The cell lines are not listed as mis-identified in that database

Human research participants

Policy information about studies involving human research participants

Population characteristics	The human research participants were Han Chinese females of various ages as specified in the Supplementary Data 1
Recruitment	The participants were recruited in Fujian province, China
Ethics oversight	All donors have given informed consent and the experiments were approved by the ethics review board of the Quanzhou 2nd Affiliated Hospital and School of Biomedical Sciences, Huaqiao University.

Note that full information on the approval of the study protocol must also be provided in the manuscript.

Flow Cytometry

Plots

Confirm that:

☒ The axis labels state the marker and fluorochrome used (e.g. CD4-FITC).

☒ The axis scales are clearly visible. Include numbers along axes only for bottom left plot of group (a 'group' is an analysis of identical markers).

☒ All plots are contour plots with outliers or pseudocolor plots.

☒ A numerical value for number of cells or percentage (with statistics) is provided.

Methodology

Sample preparation	Apoptosis was detected on fresh K562 cells using Annexin V-FITC/PI Apoptosis Detection Kit (Solarbio Life Science) according to the manufacturer's instructions
Instrument	CytoFLEX S flow cytometer (Beckman Coulter)
Software	CytExpert 2.0
Cell population abundance	Not applicable to this work
Gating strategy	The gating strategy shown in the Supplementary Figure 2 was chosen based on presence of two clearly identifiable populations after Annexin V staining

☒ Tick this box to confirm that a figure exemplifying the gating strategy is provided in the Supplementary Information.
